# Evidence and Impacts of Nanoplastic Accumulation on Crop Grains

**DOI:** 10.1002/advs.202202336

**Published:** 2022-10-17

**Authors:** Meng Jiang, Binqiang Wang, Rui Ye, Ning Yu, Zhenming Xie, Yuejin Hua, Ruhong Zhou, Bing Tian, Shang Dai

**Affiliations:** ^1^ MOE Key Laboratory of Biosystems Homeostasis & Protection College of Life Sciences Zhejiang University Hangzhou 310012 P. R. China; ^2^ Hainan Institute Zhejiang University Yazhou Bay Sci‐Tech City Sanya 572025 P. R. China; ^3^ National Key Laboratory of Rice Biology Institute of Crop Sciences Zhejiang University Hangzhou 310012 P. R. China; ^4^ School of Physics Institute of Quantitative Biology Zhejiang University Hangzhou 310012 P. R. China; ^5^ Cancer Center Zhejiang University Hangzhou 310012 P. R. China

**Keywords:** accumulation, crop grains, nanoplastics, nutritional quality

## Abstract

Nanoplastics are emerging pollutants of global concern. Agricultural soil is becoming a primary sink for nanoplastics generated from plastic debris. The uptake and accumulation of nanoplastics by crops contaminate the food chain and pose unexpected risks to human health. However, whether nanoplastics can enter grains and their impact on the grains of crop grown in contaminated soil is still unknown. Here, the translocation of polystyrene nanoplastics (PS‐NPs) in crops, including peanut (*Arachis hypogaea* L.) and rice (*Oryza sativa* L.) is investigated. It is demonstrated PS‐NPs translocation from the root and accumulation in the grains at the maturation stage. The treatment with PS‐NPs (250 mg kg^−1^) increases the empty‐shell numbers of rice grain by 35.45%, thereby decreasing the seed‐setting rate of rice by 3.02%, and also decreases the average seed weight of peanuts by 3.45%. Moreover, PS‐NPs exerted adverse effects on nutritional quality, such as decreasing the content of mineral elements, amino acids, and unsaturated fatty acids. To the knowledge, this is the first report of the presence of nanoplastics in the grains of crop plants grown in soil containing nanoplastics, and the results highlight the impact of nanoplastics on the yield and nutritional quality of crop grains.

## Graphical abstract

This study demonstrates that polystyrene nanoplastics could accumulate in crop grains, and pose adverse effects on nutritional quality of crop grains. Considering the ubiquitous distribution of plastic debris, the uptake and accumulation of nanoplastics by crops can contaminate the food chain, pose negative impacts on the crop economy and also may bring unexpected risks to human health.

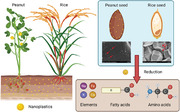

## 1 Introduction

Plastic production has constantly increased over the last 30 years,^[^ ^1^ ^]^ and the annual production of plastics has exceeded 360 million tons in 2018.^[^ ^2^ ^]^ Polystyrene (PS), which contains a high ratio of aromatic components, has become one of the most dominant and hazardous species of plastics.^[^ ^3^ ^]^ Under climate change, sunlight radiation and slow biodegradation,^[^ ^4^, ^5^, ^6^ ^]^ large plastic wastes can be fragmented into microplastics (100 nm–5 mm) and further broken down into nanoplastics (<100 nm). Plastic debris is contaminating oceans, lakes, lands, and households.^[^ ^7^, ^8^, ^9^ ^]^ Nanoplastics (NPs) are extensively distributed in the living environment, especially in the aqueous environment.^[^ ^10^, ^11^, ^12^ ^]^ PS‐NPs pose adverse effects on living organisms.^[^ ^3^ ^]^ In aquatic and terrestrial animals, PS‐NPs accumulate in organs and cells through the food chain and cause oxidative stress, inflammation, neurotoxicity, as well as neoplasia changes in metabolism and energy homeostasis.^[^ ^13^, ^14^, ^15^, ^16^ ^]^

Crops, which are important parts of the food chain, possibly take up and accumulate toxic nanoparticles from the soil. However, terrestrial ecosystems are receiving far less scientific attention with regard to nanoplastics than their aquatic counterparts. Several studies have confirmed that nanoplastics and microplastics can enter plant roots and translocate to leaves.^[^ ^17^, ^18^, ^19^, ^20^ ^]^ In wheat and lettuce, PS‐NPs enter plant tissues via a crack‐entry mode.^[^ ^21^ ^]^ Moreover, charged nanoplastics could accumulate and strongly inhibit *Arabidopsis thaliana* growth and development.^[^ ^17^ ^]^ The consumption of nanoplastic‐contaminated crop grains may pose potential risks to humans.^[^ ^22^ ^]^ Although recent studies have evaluated the entrance, distribution, and toxicity of nanoplastics in plants, very little is known about the accumulation and interactions of nanoplastics within seeds, which are the basic living component of the ecosystem at the lowest trophic level of the food chain.

As an important economic crop, rice is the staple food of over half of the world population;^[^ ^23^ ^]^ peanut is an excellent source of protein and fatty acids (FAs) and is second only to the soybean in terms of quantity and nutrition.^[^ ^24^ ^]^ The present study aimed to determine whether nanoplastics in soil could translocate into crop grains. Rice and peanut, whose seeds grow on the ground and underground, respectively, were selected as plant models. For the first time, we demonstrated that nanoplastics could accumulate in the grains of rice and peanut. Additionally, nanoplastics exerted a remarkable negative effect on the quality of crop grains. These results indicated that the overuse of plastic products in agriculture affects food safety throughout the food chain.

## 2 Results and Discussion

### 2.1 Characterization of PS‐NPs

**Figure 1:**
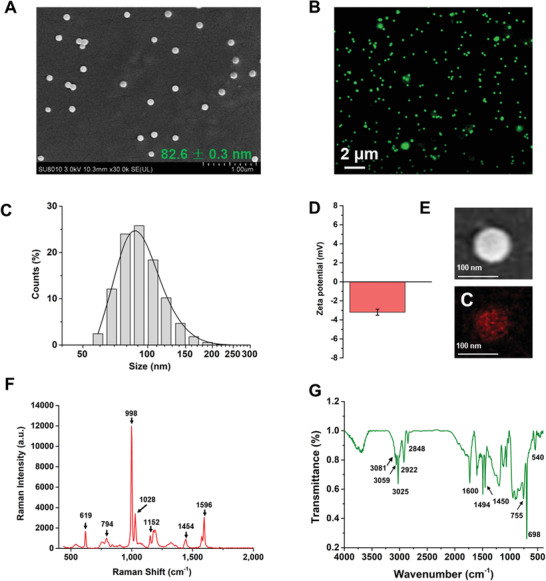
Characterization of PS‐NPs. A) SEM images of PS‐NPs. B) Fluorescence images of PS‐NPs. C) The distribution of hydrodynamic diameter of PS‐NPs was measured by DLS at 25 °C and pH 7.0. D) Zeta potential of PS‐NPs in distilled water at 25 °C and pH 7.0 (data presented as mean ± SD, *n* = 3). E) Elemental mappings of PS‐NPs in the SEM image; the red dots are the signals of carbon element. F) Raman spectra of PS‐NPs. G) FTIR spectra of PS‐NPs.

As shown in the scanning electron microscope (SEM) image (**Figure** **1**A), the PS‐NPs were nearly monodispersed with spherical morphology. The green fluorescently labeled PS‐NPs were detected at 505 nm excitation and 515 nm emission wavelengths under the confocal laser scanning microscope (Figure 1B). The average size of PS‐NPs was 82.6 ± 0.3 nm, as detected by using SEM. The hydrodynamic particle size and zeta potential of PS‐NPs were further measured using the Zetasizer Nano ZS. As shown in Figure 1C, the PS‐NPs were highly monodispersed, and the size of the highest percentage of PS‐NPs (25.8%) was 91.3 nm. The zeta potential value (−3.21 ± 0.17 mV) of PS‐NPs indicated the presence of a slight negative charge on the surface of nanoplastics (Figure 1D). The analysis of PS‐NPs using SEM‐energy‐dispersive X‐ray spectrum (EDS) revealed the presence of carbon element in polystyrene (C_8_H_8_)_*n*_ (Figure 1E). Then, Raman spectra of the PS‐NPs were collected from 500 to 2000 cm^−1^. As illustrated in Figure 1F, the intensity of the *ν*12 C‐C‐C ring bending mode of PS at 998 cm^−1^ was observed, and the characteristic PS bands were also clearly observed at 619, 794, 1028, 1152, 1454, and 1596 cm^−1^, which correspond to the *ν*6b radial ring‐stretching, *ν*1 symmetric ring‐stretching, *ν*18a tangential C‐H bending, *ν*15, *ν*19b or *δ*(CH_2_), and *ν*12 C‐C stretching mode, respectively.^[^ ^25^ ^]^ In Fourier‐transform infrared spectroscopy (FTIR) spectra, the main peaks at 2848, 2922, 3025, 3059, and 3081 cm^−1^ were observed in the PS‐NPs (Figure 1G), and these peaks were attributed to the stretching vibration of C—H bonds in the aromatic rings and main chains.^[^ ^26^ ^]^ The peaks corresponding to the C—H deformation and skeleton structure vibration were also detected at 540, 698, 755 1450, 1498, and 1600 cm^−1^ in PS‐NPs (Figure 1G). Moreover, the PS‐NPs were stable in water without the release of fluorescent dyes or additives after storage for 1 year (Figure S1, Supporting Information).

### 2.2 Nanoplastic Accumulation in Crop Grains

**Figure 2:**
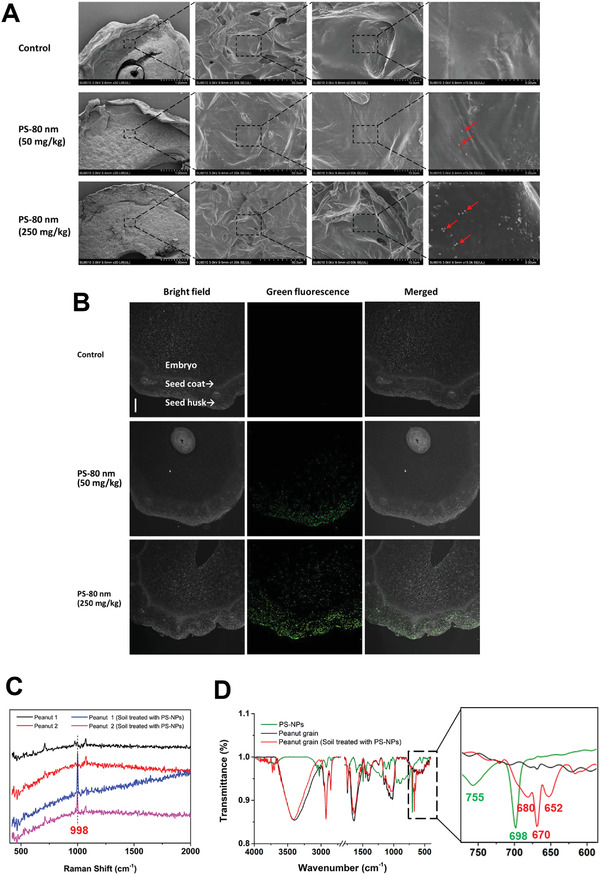
Accumulation of fluorescently labeled PS beads (80 nm) in peanut grains. A) SEM images of 80 nm PS bead localized in the peanut grain. Red arrows indicate the nanoplastics. B) Bright field images showing the transverse sections of grain from a peanut plant treated with 80 nm fluorescently labeled PS beads. Green fluorescence images of peanut grain transverse sections were observed using the green channel to detect fluorescently labeled PS beads. Scale bars, 500 µm. C) Raman spectra of peanut grains exposed to 0 and 250 mg kg^−1^ PS‐NPs (each in two samples). D) FTIR spectra of PS‐NPs (green) and peanut grains exposed to 0 (black) and 250 mg kg^−1^ (red) PS‐NPs. The right panel shows a close‐up view of the 600–750 cm^−1^ wave band.

During the uptake and transportation of nanoplastics in crops, nanoplastic polymeric beads encounter numerous physiological and chemical obstacles, which constitute the size‐exclusion limits (SELs).^[^ ^27^ ^]^ The structure and width of the obstacles vary with the environmental conditions, crop species, and growth stages. Large nanoparticles (>100 nm) are generally taken up by plants despite having plant roots with small SELs (<20 nm).^[^ ^28^ ^]^ Growing underground, peanut grains are potentially more vulnerable to environmental threats in soils. The roots of various plants absorb and accumulate microparticles and nanoparticles.^[^ ^21^, ^29^, ^30^, ^31^ ^]^ After 125 days of treatment with PS‐NPs, the amount of PS‐80 nm that accumulated in peanut roots under the treatment of 250 mg kg^−1^ PS‐80 nm in the soil was much more than that under 50 mg kg^−1^ treatment, as observed in a section of peanut root by SEM (Figure S2, Supporting Information). Moreover, many PS beads (80 nm) were observed in nanoplastics‐treated peanut grains compared with the control, as shown by SEM and fluorescence imaging (**Figure** **2**A,B; and Figures S3 and S4, Supporting Information). Raman spectra of the nanoplastics‐treated peanut grains demonstrated the presence of an obvious characteristic peak (998 cm^−1^) (Figure 2C), which corresponded to the *ν*12 C‐C‐C ring bending mode of PS. Moreover, FTIR spectra showed that 680, 670, and 652 cm^−1^ with strong intensities appeared in nanoplastics‐treated peanut grains (Figure 2D), which represented the CH out‐of‐plane bending vibrations (600–900 cm^−1^).^[^ ^32^ ^]^

**Figure 3:**
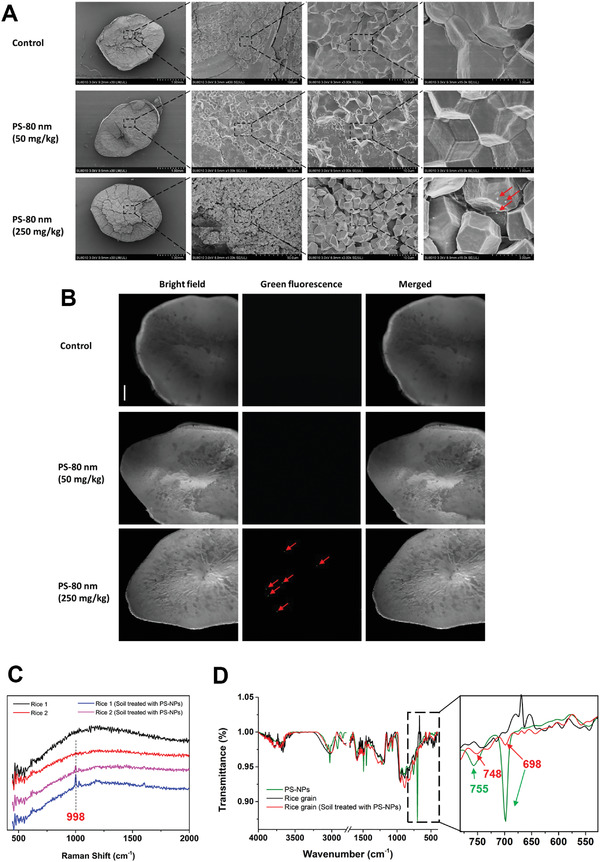
Accumulation of fluorescently labeled PS beads (80 nm) in rice grains. A) SEM images of 80 nm PS bead localization in the rice grain. Red arrows indicate nanoplastics. B) Bright field images showing transverse sections of grain from a rice plant treated with 80 nm fluorescently labeled PS beads. Green fluorescence images of transverse sections of a rice grain were observed using the green channel to detect the fluorescently labeled PS beads. Red arrows indicate nanoplastics. Scale bars, 500 µm. C) Raman spectra of rice grains exposed to 0 and 250 mg kg^−1^ PS‐NPs (each in two samples). D) FTIR spectra of PS‐NPs (green) and rice grains exposed to 0 (black) and 250 mg kg^−1^ (red) PS‐NPs. The right panel shows a close‐up view of the 550–750 cm^−1^ wave band.

For rice, a recent study has shown that PS‐NPs accumulate in the vascular systems of roots, stems, and leaves, especially root stele, stem vascular bundles, and leaf veins of rice seedlings, indicating that apoplastic transport may be the main pathway for the uptake and translocation of PS in rice tissues.^[^ ^20^ ^]^ Our results confirmed that PS‐NPs can accumulate in the roots and leaves of adult rice plants (Figures S5 and S6, Supporting Information). In adult rice plants, PS‐NPs were also detected in spikes by SEM and laser confocal microscope (Figure S7, Supporting Information), whose germination is an important factor affecting the yield and processing quality of rice grain.^[^ ^33^ ^]^ Ten days after flowering, the husk of immature rice grains in the grouting period was removed, and the liquid starch was subjected to fluorescence imaging. Nanoplastics entered the grain at the early grain‐setting stage of rice (Figure S8, Supporting Information). After the grouting period, PS‐NPs were accumulated in the starch granule of the mature and solid rice grain, as determined by SEM and fluorescence imaging (**Figure** **3**A,B; and Figures S9 and S10, Supporting Information). Raman spectra of the nanoplastic‐treated rice grains showed the characteristic peak (998 cm^−1^) of PS (Figure 3C). FTIR spectra of the nanoplastics‐treated rice grains showed 698, and 748 cm^−1^ shifted close to 755 cm^−1^, which correspond to the C—H deformation and skeleton structure vibration of PS (Figure 3D).

Previous studies proved that nanoplastics could translocate across cell membranes.^[^ ^34^ ^]^ There are a large number of endosperm cells and embryo cells in peanut and rice grains, and the endocytosis of nanoplastics may occur in these cells. Here, we further performed coarse‐grained molecular dynamics simulations to investigate the interaction of the PS‐NPs with plant cell membranes as well as the possible endocytosis process. The modeling details of the PS‐NPs and membrane lipid bilayer were provided in the Experimental Section (Supporting Information). As shown in Figure S11 (Supporting Information), after the initial random motion, the PS‐NPs was attached to the membrane and gradually inserted into the membrane due to the van der Waals interaction between the PS‐NPs and lipid membrane. Then, the PS‐NPs was partially encapsulated by the lipid layer and finally endocytosed (Figure S11, Supporting Information), indicating that the PS‐NPs may enter the cells in grains by endocytosis. Therefore, the present results experimentally and theoretically demonstrated that PS‐NPs (80 nm) from crop roots could translocate to and accumulate in the grains.

### 2.3 Changes in Physiological Parameters of Crop Grains Exposed to PS‐NPs

**Figure 4:**
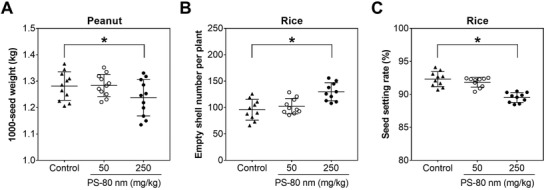
Physiological responses of peanut and rice grains upon exposure to PS treatments. A–C) represent the 1000‐seed weight of peanut grains, and empty shell number per plant and seed setting rate of rice grains under PS treatments with different concentrations, respectively (data presented as mean ± SD, *n* = 10, *P*‐values are calculated using Student's *t*‐test, *P < 0.05).

The phenotypic images of nanoplastic‐treated plants showed that nanoplastic exposure caused no significant impact on plant height and tiller number per plant of peanut and rice (Figures S12 and S13, Supporting Information). Moreover, nanoplastic exposure at different concentrations had no significant impact on the yield per plant of peanut (Figure S12, Supporting Information) and the panicle length (Figure S13, Supporting Information), 1000‐seed weight and total grain number per plant of rice (Figures S13 and S14, Supporting Information). The presence of 50 mg kg^−1^ PS‐NPs had no impact on the 1000‐seed weight of peanut; 250 mg kg^−1^ PS‐NPs decreased the 1000‐seed weight of peanut by 3.45% relative to the control (**Figure** **4**A). Similar results were evident in rice empty‐shell number per plant and seed‐setting rate. The rice empty‐shell number per plant increased by 35.45% compared with the control, and the seed‐setting rate decreased by 3.02% in the presence of 250 mg kg^−1^ PS‐80 nm compared with the control (Figure 4B,C). Metal‐based nanoparticles such as silver (Ag), cerium oxide (CeO_2_), zinc oxide (ZnO), and copper oxide (CuO) nanoparticles could be absorbed and accumulate in crop grains, thereby exerting adverse effects.^[^ ^35^, ^36^, ^37^, ^38^ ^]^ Our results suggested that PS‐NPs could pose adverse effects on crop grains, which may decrease the crop yield.

### 2.4 Effect of PS‐NPs on Mineral Elements Homeostasis in Grains

**Figure 5:**
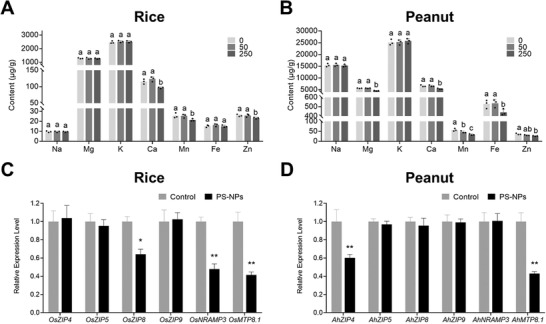
Effect of PS‐NPs on mineral elements homeostasis in rice and peanut grains. Content of mineral elements in rice A) and peanut B) grains exposed to 0, 50, or 250 mg kg^−1^ PS‐NPs (data presented as mean ± SD, *n* = 3, *P*‐values are calculated using Bonferroni Post‐tests, different letters above the error bars indicate the significance across groups, *P* < 0.05). The gene expressions of metal ion transport‐related genes in rice C) and peanut D) grains exposed to 0 (control) or 250 mg kg^−1^ PS‐NPs (data presented as mean ± SD, *n* = 3, *P*‐values are calculated using Student's *t*‐test, *P < 0.05, **P < 0.01).

We investigated the mineral concentrations in peanut and rice grains exposed to PS‐NPs. **Figure** **5**A,B illustrated that 50 mg kg^−1^ PS‐80 nm treatment reduced 18.74% of Mn content in peanut grains but did not modified other mineral elements in peanut or rice grains. In the presence of 250 mg kg^−1^ PS‐80 nm, the contents of Ca, Mn, and Zn in rice grains decreased by 16.33%, 16.86%, and 9.94%, respectively, compared with those of the control (Figure 5A). The contents of Mg, Ca, Mn, Fe, and Zn in 250 mg kg^−1^ PS‐80 nm treated peanut grains decreased by 18.83%, 19.01%, 41.66%, 16.85%, and 26.95%, respectively, compared with those in the control (Figure 5B). Previous studies also showed that nanoplastics could disturb the equilibrium of metal elements in wheat and lettuces; in particular, they reduce the contents of Fe, Mn, and Zn in roots and shoots.^[^ ^19^, ^39^ ^]^ The imbalance among trace elements in tissues possibly further influences the crop grains’ mineral element content, and metal cation transports in grains may also affect the homeostasis of trace elements. For the metal cation transport in rice, OsZIP4,^[^ ^40^ ^]^ OsZIP5,^[^ ^41^ ^]^ OsZIP8,^[^ ^42^ ^]^ and OsZIP9^[^ ^41^ ^]^ are zinc‐regulated zinc transporters. OsNRAMP3^[^ ^43^ ^]^ and OsMTP8.1^[^ ^44^ ^]^ are the vascular bundles‐specific Mn transporter and cation diffusion facilitator‐related Mn transporter in rice, respectively. The homologous genes of these rice genes in peanuts were analyzed and the changes in their gene expression were measured. The results showed the gene expressions of *OsZIP8*, *OsNRAMP3*, and *OsMTP8.1* in rice exposed to PS‐NPs were down‐regulated to 0.64‐, 0.48‐, and 0.41‐fold, respectively, compared to the control (Figure 5C). The Zn and Mn transporter homologous genes (*AhZIP4* and *AhMTP8.1*) in peanuts following PS‐NPs exposure were down‐regulated to 0.60‐ and 0.43‐fold, respectively, compared with the control (Figure 5D), indicating that the decreased in Zn and Mn transporters induced by PS‐NPs may lead to respective metal ion reduction in peanut grains. Overall, our results indicated that PS‐NPs affected the balance of trace elements in peanuts and rice grains.

### 2.5 Effect of PS‐NPs on Fatty Acid Homeostasis in Grains

**Figure 6:**
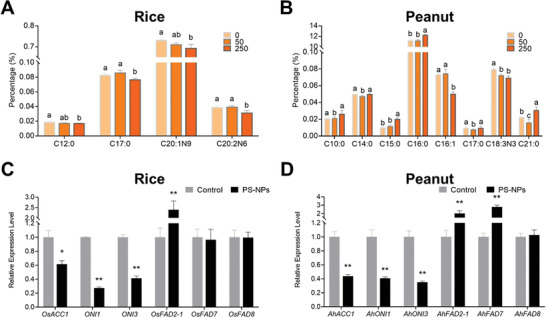
Effect of PS‐NPs on FA homeostasis in peanut and rice grains. Content of FAs in rice A) and peanut B) grains exposed to 0, 50, or 250 mg kg^−1^ PS‐NPs (data presented as mean ± SD, *n* = 3, *P*‐values are calculated using Bonferroni Post‐tests, different letters above the error bars indicate the significance across groups, *P* < 0.05). The gene expression of FAs synthesis and metabolism‐related genes in rice C) and peanut D) grains exposed to 0 (control) or 250 mg kg^−1^ PS‐NPs (data presented as mean ± SD, *n* = 3, *P*‐values are calculated using Student's *t*‐test, *P < 0.05, **P < 0.01).

Fatty acids (FAs) are important energy sources and essential components of membrane lipids that play crucial roles in the overall stress response mechanism of a plant.^[^ ^45^, ^46^ ^]^ In polished or milled rice, the FA concentration is 0.3–1.0% of the grain on average.^[^ ^47^ ^]^ Meanwhile, in peanuts, FAs account for 40–50% of the grains by weight.^[^ ^24^ ^]^ About 80% of FAs in peanuts are unsaturated FAs (UFA), and are mostly oleic and linoleic acids.^[^ ^24^ ^]^ The FA contents were measured in PS‐NPs‐treated peanut and rice grains to investigate potential alterations in the FA profiles (Tables S2 and S3, Supporting Information). Exposure to 50 mg kg^−1^ PS‐NPs had little effect on the FA profiles of rice grains (**Figure** **6**A). However, exposure to 250 mg kg^−1^ PS‐NPs significantly lowered the contents of UFAs in rice grains, including the C20:1N9 and C20:2N6, and also decreased the contents of saturated FAs such as C12:0 and C17:0 (Figure 6A).

Similarly, exposure to 50 mg kg^−1^ PS‐80 nm had no significant influence on major FAs in peanut grains, and only the relative contents of C14:0, C17:0, C18:3N3, and C21:0 showed slight changes (Figure 6B; and Table S3, Supporting Information). Meanwhile, exposure to 250 mg kg^−1^ PS‐80 nm significantly lowered the contents of the UFAs C16:1 and C18:3N3 but increased the contents of several saturated FAs, namely, C10:0, C15:0, and C16:0 (Figure 6B). Abiotic stressors reduce the contents of UFAs and convert UFAs to saturated FAs in plants.^[^ ^47^, ^48^ ^]^ Reactive oxygen species (ROS) accumulation induced by exposure to nanoplastics has been found in various plant species.^[^ ^49^, ^50^ ^]^ Therefore, the ROS induced by nanoplastics may be an important factor that decreased the UFAs in crop grains. Moreover, previous studies demonstrated that nanoparticles could also influence the gene expression of plants.^[^ ^51^, ^52^ ^]^ Thus, PS‐NPs possibly influence the gene expressions of FA synthesis‐related genes in grains. For FA synthesis and metabolism, acetyl‐CoA carboxylase OsACC1,^[^ ^53^ ^]^ FA elongase ONI1,^[^ ^54^ ^]^ long‐chain FA *ω*‐alcohol dehydrogenase ONI3,^[^ ^55^ ^]^ *ω*‐6 FA desaturase OsFAD2‐1,^[^ ^56^ ^]^ *ω*‐3 fatty acid desaturase OsFAD7,^[^ ^57^ ^]^ and OsFAD8^[^ ^58^ ^]^ play their respective roles in rice. We analyzed the gene expression changes of these homologous genes in rice and peanut. The gene expressions of *OsACC1*, *ONI1*, and *ONI3* in rice grains exposed to PS‐NPs were down‐regulated to 0.61‐, 0.27‐, and 0.41‐fold compared with the control, respectively; while the expression of *OsFAD2‐1* was increased by 2.4‐fold compared with the control (Figure 6C). For peanut grains, the gene expression levels of the homologous genes (*AhACC1*, *AhONI1*, and *AhONI3*) in peanut grains exposed to PS‐NPs were decreased to 0.44‐, 0.41‐, and 0.35‐fold (Figure 6D) compared with the control, respectively. The expressions of *AhFAD2‐1* and *AhFAD7* were up‐regulated by 2.0‐ and 2.8‐fold (Figure 6D), respectively. The *ACC1* gene homologue (*OsACC1* in rice and *AhACC1* in peanut) is a rate‐limiting enzyme in the fatty acid synthesis pathway.^[^ ^59^ ^]^ The down‐regulation of *ACC1, ONI1*, and *ONI3* induced by PS‐NPs may affect the synthesis of FAs. UFAs, which are generated by FA desaturase dehydrogenation, have an antioxidative effect due to their unsaturated double bonds.^[^ ^60^ ^]^ The increasing gene expression levels of FA desaturase (*OsFAD2‐1, AhFAD2‐1*, and *AhFAD7*) may be due to the response to oxidative stress caused by nanoplastics.

Cerium oxide nanoparticles could modify FA contents in rice.^[^ ^61^ ^]^ Metal oxide nanoparticles, such as copper oxide (CuO), iron oxide (Fe_2_O_3_), and titanium oxide (TiO_2_) nanoparticles could change the FA profiles in peanut grains.^[^ ^24^ ^]^ Different from metal‐oxide nanoparticles, nanoplastics are hydrophobic and not bioreducible. Our results revealed that PS‐NPs could disturb FA synthesis and storage in rice and peanut grains.

### 2.6 Effect of PS‐NPs on Amino Acid Homeostasis in Grains

**Figure 7:**
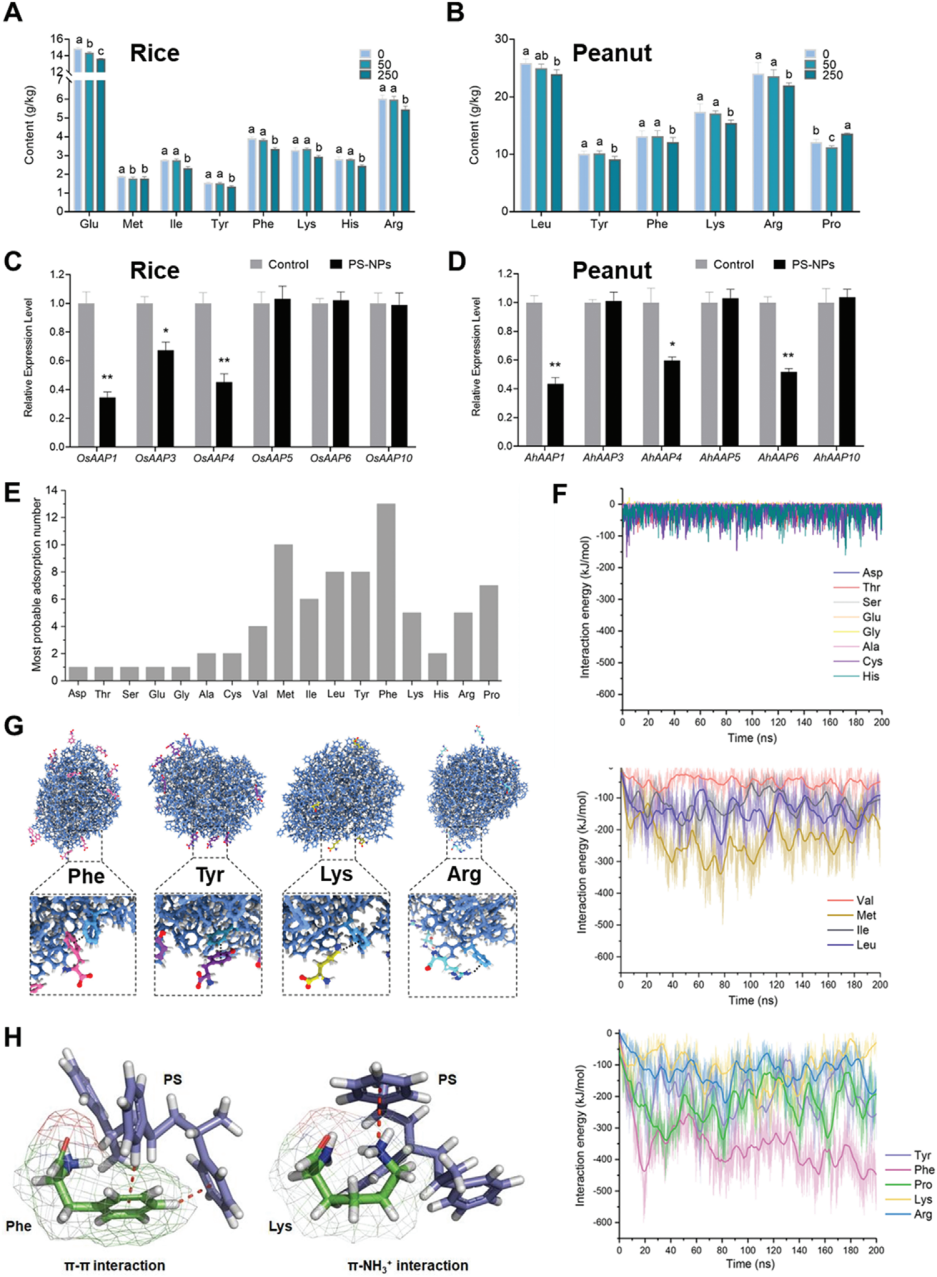
Effect of PS‐NPs on amino acid homeostasis in rice and peanut grains. Content of amino acids in rice A) and peanut B) grains exposed to 0, 50, or 250 mg kg^−1^ PS‐NPs (data presented as mean ± SD, *n* = 3, *P*‐values are calculated using Bonferroni Post‐tests, different letters above the error bars indicate the significance across groups, *P* < 0.05). The gene expressions of amino acid synthesis‐ and metabolism‐related genes in rice C) and peanut D) grains exposed to 0 or 250 mg kg^−1^ PS‐NPs (data presented as mean ± SD, *n* = 3, *P*‐values are calculated using Student's *t*‐test, *P < 0.05, **P < 0.01). E) The most probable adsorption numbers of amino acids on PS‐NPs. F) The interaction energies (including van der Waals and Coulombic energies) between amino acids and PS‐NPs. G) The snapshots of the adsorption conformation of amino acids on PS‐NPs. The PS‐NPs, Phe, Tyr, Lys, and Arg are colored by blue, pink, purple, yellow, and bright blue, respectively. H) Molecular docking model of PS and amino acids (Phe and Lys).

The amino acid contents of peanut and rice grains after exposure to different concentrations of nanoplastics are shown in Tables S4 and S5 (Supporting Information). The total amino acid content under 50 mg kg^−1^ PS‐NPs treatment had no significant changes, whereas 250 mg kg^−1^ of PS‐NPs exposure significantly lowered the total amino acid content in rice compared with the control (Tables S4 and S5, Supporting Information). Under 250 mg kg^−1^ of PS‐80 nm treatment, the nanoplastics in the soil led to an imbalance among free amino acids in rice. The contents of Glu, Met, Ile, Tyr, Phe, Lys, His, and Arg decreased by 8.32%, 5.82%, 15.85%, 12.95%, 14.67%, 10.73%, 12.77%, and 9.44%, respectively (**Figure** **7**A). Similarly, under 250 mg kg^−1^ of PS‐80 nm treatment, the contents of Leu, Tyr, Phe, Lys, and Arg in peanut decreased by 7.26%, 9.58%, 7.77%, 11.39%, and 8.58% compared with the control, respectively (Figure 7B). A common finding was that the contents of Tyr, Phe, Lys, and Arg decreased in peanut and rice grains under 250 mg kg^−1^ of PS‐NPs treatment. Previous studies demonstrated that nanoplastics in constructed wetlands disturb nitrogen uptake by plants, which may lead to the decline in the amino acid content of crop grains.^[^ ^62^ ^]^

For amino acid transport in rice, OsAAP1,^[^ ^63^ ^]^ OsAAP3,^[^ ^64^ ^]^ OsAAP4,^[^ ^65^ ^]^ OsAAP5,^[^ ^66^ ^]^ OsAAP6,^[^ ^67^ ^]^ and OsAAP10^[^ ^68^ ^]^ are responsible for amino acid uptake and reallocation. Gene expression levels of homologous genes of these transporters in rice and peanut were analyzed. The gene expression levels of *OsAAP1*, *OsAAP3*, and *OsAAP4* in rice with PS‐NPs exposure was decreased to 0.35‐, 0.67‐, and 0.45‐fold, respectively, compared with the control (Figure 7C). The gene expression levels of *AhAAP1*, *AhAAP4*, and *AhAAP6* in peanuts exposed to PS‐NPs decreased to 0.43‐, 0.60‐, and 0.35‐fold, respectively, compared with the control (Figure 7D). These results indicated that the decrease in amino acid transporters induced by PS‐NPs may reduce the amount of amino acid in grains.

Moreover, PS‐NPs have a large number of benzene rings, which may form *π*–*π* or cation–*π* interactions with aromatic hydrocarbons and ionic compounds.^[^ ^69^, ^70^ ^]^ Such specific interactions may lead to divergent adsorption behaviors of different amino acids by PS‐NPs, which may be part of the reason for the unbalanced reduction of amino acids in grains. To better understand the mechanism underlying the interaction between the PS‐NPs and amino acids, we further performed all‐atom molecular dynamics simulations. For each type of amino acid (total of 17 types), 20 amino acid molecules were placed at least 15 Å from the PS‐NPs (Figure S15, Supporting Information), and a 200 ns molecular dynamics simulation was performed. We first studied the distribution of amino acids adsorbed on the PS‐NPs (Figure S16, Supporting Information) and then calculated the most probable adsorption number for each type of amino acid (Figure 7E). Met, Ile, Leu, Tyr, Phe, Lys, Arg, and Pro had higher adsorption propensity, suggesting their stronger binding strength with PS‐NPs. Furthermore, the interactions between these amino acids and PS‐NPs were calculated and found to be stronger than those between PS‐NPs and other amino acids (Figure 7F). Figure 7G showed that Phe and Tyr can form *π*–*π* stacking interactions with the benzene rings in PS‐NPs, making them more inclined to bind to the PS‐NPs. The large hydrophobic side chains of Lys and Arg also showed strong interactions with PS‐NPs (Figure 7G). In addition, the ionized amino groups of the side chains can form cation–*π* interaction with the benzene rings of PS‐NPs, which also contributed to the enhanced adsorption of Lys and Arg (Figure 7G). Results of molecular docking also showed that the benzene rings in PS formed “edge‐to‐face” *π*–*π* interaction with benzene rings in Phe, and interacted with the ionized amino group of the side chain in Lys to form cation–*π* interaction (Figure 7H), which is consistent with the result of molecular dynamics simulations. These simulation results are quite consistent with the experimental results of amino acids adsorbed onto PS‐NPs (Figure S17, Supporting Information). The amino acids in grains mostly originate from external nutrient transport,^[^ ^71^ ^]^ and the binding effect by root‐accumulated PS‐NPs with these amino acids in plant tissues may decrease the content of amino acids in grains. These results indicated that nanoplastics in soil affected the amino acid content and metabolism in grains of rice and peanut, which may be due to the decreased amino acid transporters induced by PS‐NPs and the effect of amino acid binding by PS‐NPs.

### 2.7 Exposure and Environmental Implications

Plastic contamination is a global issue that may cause crop food economy and safety concerns. This study showed the accumulation and impacts of nanoplastics on grain upon exposure of crop roots to PS‐NPs. Once nanoplastics enter plant roots, they can transfer into different organs, and finally into grains. Furthermore, the presence of nanoplastics in crop grains could increase the direct exposure of humans to nanoplastics. This possibility is of particular concern, especially in the case of rice cultivated in paddy soil systems containing nanoplastics that come from irrigation water and nutrient‐rich fertilizer solutions.

Considering their ubiquitous distribution, the potential ecological risks of nanoplastics, which may impair the crop quality and nutrient supply, need to be investigated. Furthermore, previous studies have shown that PS‐NPs have high surface‐to‐volume and surface hydrophobicity and could serve as vectors for a range of environmental contaminants, such as herbicides, pesticides, antibiotics, and heavy metals, thereby facilitating the accumulation of contaminants in crop grains. Thus, the entry of nanoplastic into the grains may accompany other hazards, which pose a greater threat to human health than the nanoplastics themselves. Moreover, the direct effects (nanoplastic internalization) and indirect effects (soil structure effects and microbial effects) may pose multiple impacts on grains of different plants, which should be further studied.

Our experiments were performed using PS‐NPs to study the uptake process of nanoplastics and their effects on crop quality. However, various types of nanoplastics exist in the environment, and nanoplastics comprising different materials may have different influences on crop plants and their grains. Future research should consider using nanoplastics made of different materials. It remains challenging to conclude that the experimental exposure treatments performed in this study are likely to mimic environmental conditions in terms of plastic contamination. The available analytical methods do not yet permit the accurate detection of nanosized and even sub‐micrometer‐sized plastic particles in the environment. Inspired by these findings, future studies can focus on how PS‐NPs interact with the substances in grains, as well as the potential health risks caused by the consumption of polluted grains.

## 3 Experimental Section

### Characterization of PS Nanoplastics

Stock solutions of plain fluorescently labeled PS‐NPs (without functionalization) were purchased from Da'e Scientific Co., Ltd. (Tianjin, China). They were provided as suspensions in ultrapure water with a concentration of 10 mg mL^−1^. The nominal size of PS beads was ≈80 nm. And the actual size of the PS bead was confirmed using SEM. The green fluorescently labeled PS‐NPs can be detected at 505 nm excitation and 515 nm emission wavelengths under the confocal laser scanning microscope (Zeiss LSM510). With respect to the leaching ability of fluorescent agents or additives from fluorescently labeled PS‐NPs, a sample solution (1 mL) of fluorescently labeled PS‐NPs stored in ultrapure water for 1 year was centrifuged at 10 000 × g for 5 min, then the precipitate and supernatant was monitored at 505 nm excitation and was also analyzed using Raman spectra.

Size distribution and surface charge of the PS‐NPs were measured by Dynamic light scattering (DLS) and zeta potential analysis using a laser Doppler anemometer (Zetasizer Nano ZS; Malvern Instruments, Malvern, UK) with a He—Ne laser beam at a wavelength of 632.8 nm in 25 °C.^[^ ^34^ ^]^ Briefly, 100 µL of PS‐NPs solution (10 mg mL^−1^) was diluted to 1 mL with deionized water to a final concentration (1 mg mL^−1^). An electric field of 150 mV was applied to observe the electrophoretic velocity of the particles.

The EDS was recorded by focusing the electron beam onto a region of the sample surface to perform elemental analysis of the test samples in an area scan mode.^[^ ^72^ ^]^

Raman spectroscopy measurements are performed as previously described.^[^ ^73^ ^]^ The crop grains were dried by lyophilization. Raman spectra were collected using a Raman spectrometer (Hooke Instruments Ltd., HOOKE P300) with a 785 nm excitation light. The spectrometer can provide Raman spectra in the range of 200 to 3000 cm^−1^ with a spectral resolution of 5 cm^−1^. The spectral measurements were conducted with a 10 s exposure time and laser power of 5 mW. The spectra data were collected and analyzed; the Raman spectra represented in this study were baseline corrected.

For FTIR analysis, the PS‐NPs and crop grains were dried by lyophilization. Dried samples were crushed with KBr in a mortar at a ratio of 1:100 w/w. The pressed pellet was recovered with a clip and immediately analyzed in the region of 4000–400 cm^−1^ at a resolution of ≈2 cm^−1^ over 1800 scans using Nicolet 5700 FTIR spectrometer (Thermo Nicolet Co., Madison, WI).

### Growth Condition and Plant Material

The loamy soil was collected from the experimental farm of Zhijiang Seed Tec. Ltd., Zhejiang, China. The chemical and physical characteristics of the soil are displayed in Table S1 (Supporting Information). After drying, the soil was sieved through a 2 mm mesh. PS beads (80 nm) were fully mixed with soil to reach final contents of 50 or 250 mg kg^−1^. The PS beads‐modified soils in pots were stabilized for two days. PS beads‐free repeats were utilized as controls. There were ten pots in each treatment.

The seeds of peanut (*Arachis hypogaea* L.) and rice (*Oryza sativa* L.) were selected with almost uniform size in the study. The seeds were sterilized by treatment for 5 min with a 10% v/v NaClO solution, and then washed with distilled water three times to eliminate the remaining solution of NaClO. The peanut seeds were kept in distilled water for 4 h at 50 °C, and the seeds of rice were soaked in 25 °C distilled water for 40 h. Then all the seeds were moved to wet filter paper and kept at 25 °C to prompt germination.

Every five seeds were germinated in each pot for 2 weeks, and then one selected seedling with uniform size was planted in each pot. In order to make the two species grow better, moist soil was used when growing rice and fluffy soil was used when growing peanuts. The plants of peanut and rice were planted in the growth chamber (12 h light at 28 °C/12 h dark at 25 °C) with 75% relative humidity for 125 and 150 days, respectively. Samples were gathered for the phenotypic, physiological, and biochemical analysis after the cultivation. The empty shell number, 1000‐seed weight, and full grain number of rice were measured as previously described.^[^ ^74^ ^]^ Total grain number per plant = full grain number per plant + empty shell number per plant; Seed setting rate (%) = full grain number per plant/total grain number per plant × 100; Yield per plant (g) = full grain number per plant × 1000‐seed weight /1000. Ten replicates were shown for plant height and other phenotypic analysis, and from them three replicates selected randomly were shown for physiological, and biochemical analysis. These tests were repeated independently.

### SEM

The samples from grains, leaves, and roots were collected in liquid nitrogen (peanut grains and roots of plants were washed several times in distilled water for removing soil and surface nanoplastics before liquid nitrogen freezing), then freeze‐dried for about 20 h and coated for 60 s with gold (about a 1 nm thickness of gold). The samples were finally detected by a scanning electron microscope (SEM) SU8010 (Hitachi, Tokyo, Japan).^[^ ^75^ ^]^

### Measurement of Mineral Accumulation

Mineral accumulation in crop grains was measured according to the previous method.^[^ ^76^ ^]^ Briefly, crop grains were dried for 30 min at 105 °C and kept for 24 h at 60 °C. Crop grains were ground into powder. Crop grain samples were digested at 160 °C for 45 min with a 200 mg sample in 6 mL HNO_3_. The digested solution was concentrated for 3 h at 140 °C with ≈1 mL solution left, and finally diluted to 50 mL with distilled water. The mineral accumulation was evaluated by an atomic absorption spectrometer AA‐7000 (Shimadzu, Tokyo, Japan).

### Measurement of Fatty Acid Content

The concentrations of fatty acids were measured as previously described.^[^ ^24^ ^]^ Grains were freeze‐dried for 2 days, and were pulverized to powder. About 100 mg samples were added with 1 mL n‐hexane, 4 mL chloroacetic methanol, and 1 mL undecanoic acid methyl ester, and then kept at 80 °C for 150 min. After cooling to room temperature, potassium carbonate (5 mL, 7%) was dripped into the blend. After centrifuging for 5 min at 1000 × *g*, the supernatant was filtered through a 0.22 µm filter membrane. The contents of fatty acids were finally evaluated by using gas chromatography (GC) Agilent 6890 (Agilent, CA) with the flame ionization detector (FID). Validation parameters of GC‐FID are as follows: the correlation coefficient (r^2^) of the standard curve was 0.9986–0.9997, the limit of detection (LOD) was 0.085 µg, and the limit quantitation (LOQ) was 0.25 µg.

### Free Amino Acids Profile

The measurement of free amino acids was performed according to Song et al.^[^ ^77^ ^]^ A solution of 2% w/v sulfosalicylic acid was added to about 100 mg of freeze‐dried grains. The mixture was kept for 2 h at room temperature with 200 × *g*, then centrifuged for 15 min at 12 000 × *g*. After being filtered by a 0.22 µm membrane, the supernatant was arranged for the analysis of free amino acids by a free amino acid automatic instrument L‐8900 (Hitachi, Tokyo, Japan).

### Assay of Gene Expression

The relative gene expression level was measured according to the protocol of the previous study.^[^ ^78^, ^79^ ^]^ The RNAprep Pure Plant kit (Tiangen, Beijing, China) and HiScript III 1st Strand cDNA Synthesis kit (Vazyme Biotech Co., Ltd., Nanjing, China) were used to extract and reverse transcribe the total RNA from rice and peanut grains, respectively. AceQ qPCR SYBR Green Master Mix (Vazyme, Nanjing, China) was used to apply quantitative real‐time PCR (qRT‐PCR). The qRT‐PCR primers are listed in Additional file1. The housekeeping genes: UBQ5 gene (Accession No: Os06g0650100) in rice and the ELF1B gene (Accession No: EE126175) in peanut, were used as internal controls, respectively. The level of gene expression was presented as the value of 2^−ΔΔCt^.

### All‐Atom Molecular Dynamics Simulation

A PS‐NPs model containing 20 polystyrene chains, with each chain possessing 20 styrene monomers (PS20, neutrally charged) was first constructed. For each type of amino acid (total of 17 types), the PS‐NPs are placed in the center of a solvation box (with the size of 10 ×10 × 10 nm^3^), and multiple amino acid molecules (20 was used in this case) with the same type (to enhance sampling statistics) were placed at least 15 Å from the PS‐NPs (Figure S15A, Supporting Information). These systems were solvated in water and neutralized with sodium and chlorine ions, yielding a physiological ionic strength of 150 mm. To better investigate the distribution of amino acids around the PS‐NPs, the PS‐NPs is largely kept in the center of the box throughout the simulation by constraining the position of a benzene group near the center of PS‐NPs. Then, a 200 ns molecular dynamics simulation for each system was performed.

The CHARMM36 force field^[^ ^80^, ^81^, ^82^ ^]^ was used for amino acids and NaCl. The force field parameters of PS‐NPs were obtained from previous works.^[^ ^83^, ^84^ ^]^ The TIP3P model^[^ ^85^ ^]^ was chosen for water molecules. The periodic boundary conditions were applied in all three dimensions. The long‐range electrostatic interactions were computed with the particle mesh Ewald (PME) method,^[^ ^86^ ^]^ and the vdW interactions were treated with a smooth cutoff (cutoff distance of 1.2 nm). The LINCS algorithm was adopted to constrain the bond vibrations involving hydrogen atoms,^[^ ^87^ ^]^ allowing for a time step of 2 fs. The system temperature (T = 300 K) was controlled using the velocity‐rescaled Berendsen thermostat.^[^ ^88^ ^]^ The pressure was set to 1 atm using an isotropic Parrinello–Rahman pressostat.^[^ ^89^ ^]^ After the equilibration of the simulation system, each production run was carried out in the NPT ensemble. The all‐atom molecular dynamics simulations were conducted with the GROMACS package (version 5.1.4).^[^ ^90^ ^]^ Snapshots were rendered by the visual molecular dynamics (VMD) program and Chimera.^[^ ^91^, ^92^ ^]^

### Molecular Docking

Molecular docking was performed using AutoDock 4.2 (http://autodock.scripps.edu). The amino acids (Phe and Lys) and PS (Three unit molecule styrene) were built in PyMOL (https://pymol.org/2/). For the docking routine, the ligands were considered as flexible molecules and all rotatable bonds of the ligand were rotated in the docking software to provide the best conformation of the ligands interacting with PS‐NPs. The structure with the lowest binding free energy in the cluster was selected as the optimal docking pose.

### Statistical Analyses

The data in the tables and figures was shown as means ± standard deviation (SD) from three replicates (*n* = 3) for assays on the effects of PS‐NPs on metabolite homeostasis or ten replicates (*n* = 10) for measurements of the yield parameters of crops. For the significant difference among more than two groups, one‐way ANOVA testing followed by Bonferroni Post‐tests was carried out and the statistical significance across groups were indicated by different letters (*P* < 0.05). For the significant difference between the two groups, Students’*t*‐test was performed and statistical significance was defined as *P < 0.05, **P < 0.01. Statistical analysis was carried out using GraphPad Prism 9.0.

## Conflict of Interest

The authors declare no conflict of interest.

## Author Contributions

M.J, B.W., and R.Y. contributed equally to this work. M.J., B.T., R.Z., and S.D. were responsible for the experimental design. M.J., B.W., Z.X., and N.Y. performed the experiments. R.Y. and R.Z. performed the molecular dynamic analysis. M.J., B.T., R.Z., S.D., and Y.H. performed data analysis and drafted the manuscript. All authors read and approved the version to be published.

## Supporting information

Supporting Information 1

Supporting Information 2

## Data Availability

The data that support the findings of this study are available in the supplementary material of this article.
