# Supplementary material for: Evidence and Impacts of Nanoplastic Accumulation on Crop Grains
Source: Adv Sci (Weinh). 2022 Oct 17;9(33):2202336. doi: 10.1002/advs.202202336 (PMC9685458; doi:10.1002/advs.202202336)
Supplement: Supplementary file 1 — Supporting Information 1 [file ADVS-9-2202336-s002.pdf]

ADVANCED SCIENCE
Open Access

Supporting Information

for *Adv. Sci.*, DOI 10.1002/advs.202202336

Evidence and Impacts of Nanoplastic Accumulation on Crop Grains

Meng Jiang, Binqiang Wang, Rui Ye, Ning Yu, Zhenming Xie, Yuejin Hua, Ruhong Zhou, Bing Tian* and Shang Dai**

Evidence and impacts of nanoplastic accumulation on crop grains

Meng Jiang[1,2,3,#], Binqiang Wang[1,#], Rui Ye[1,4,#], Ning Yu[1], Zhenming Xie[1], Yuejin Hua[1], Ruhong Zhou[1,4,5,*], Bing Tian[1,5,*], Shang Dai[1,*]

[1] MOE Key Laboratory of Biosystems Homeostasis & Protection, College of Life Sciences, Zhejiang University, Hangzhou, China

[2] Hainan Institute, Zhejiang University, Sanya, China

[3] National Key Laboratory of Rice Biology, Institute of Crop Sciences, Zhejiang University, Hangzhou, China

[4] School of Physics, Institute of Quantitative Biology, Zhejiang University, Hangzhou, China

[5] Cancer Center, Zhejiang University, Hangzhou, China

[*]Corresponding author: Shang Dai, Bing Tian, Ruhong Zhou

Email: daishang@zju.edu.cn, tianbing@zju.edu.cn, rhzhou@zju.edu.cn

Author contributions

M.J., B.T., R.Z. and S.D. were responsible for the experimental design. M.J., B.W., Z.X., and N.Y. performed the experiments. R.Y., R.Z. performed the molecular dynamic analysis. M.J., B.T., R.Z., S.D. and Y.H. performed data analysis and drafted the manuscript. All authors read and approved the version to be published.

M.J, B.W., and R.Y. contributed equally to this work

The authors declare no competing interest.

Key words: nanoplastics, accumulation, crop grains, nutritional quality

Materials and methods

Coarse-grained Molecular Dynamics Simulation

We first constructed a polystyrene chain containing 100 styrene monomers (PS100, neutrally charged). Then, 273 polystyrene chains were aggregated into a nanoplastic sphere with a diameter of 20 nm (**Figure S11A and B**).

More specifically, each styrene monomer was constructed by mapping onto four interaction beads: one corresponding to the position of the backbone atoms, and the other three representing the benzene ring. This mapping corresponded to the "A-mapping" scheme developed by Rossi *et al*. that reproduced the best structural properties of polystyrene.[1]

For the plant cell membrane, three common phospholipids (POPC, POPE and POPI) in the plants were adopted[2-3] to construct the lipid bilayer with a molar ratio of 2:1:1 (POPC:POPE:POPI). [4-5] The membrane contained 10608 POPC, 5304 POPE and 5304 POPI, with the surface dimensions of 80nm × 80nm (**Figures S11A and B**). The membrane models were generated by using *insane.py* script. [6] Then, the membrane was equilibrated for 1000 ns at 300 K and 1 atm. The final membrane structure and the PS-NPs were then placed into a water box with a size of 80 nm × 80 nm × 45 nm to study their interactions.

Molecular dynamics simulations on the coarse-grained models were performed in the NPT ensemble (P = 1 atm and T = 300 K). The pressure was controlled using isotropic Parrinello-Rahman pressostat,[7] and the system temperature was maintained using a stochastic velocity rescaling thermostat.[8] The Martini force field[9] was adopted to describe the interactions of the lipid membrane and water molecules, and the parameters developed by Rossi *et al*.[1] were employed for nanoplastics. The periodic boundary conditions were applied in all directions. Short-range electrostatic and van der Waals interactions were calculated with a cut-off distance of 1.1 nm. The long-range electrostatic interactions were treated using reaction-field potential.[10] The all-atom molecular dynamics simulations were conducted with the GROMACS package (version 5.1.4).[11] Snapshots were rendered by the visual molecular

dynamics (VMD) program.[12]

Measurement of PS-binding amino acids

For measurement of PS-binding amino acids, amino acid mix solution (the content of each amino acid was 20 ng/ml) was prepared, and 1mL of mix solution was incubated with 10 μL PS nanoplastics (10 mg/mL) for 12 h. Then the mixture was centrifuged for 15 min at 20,000 × g and the supernatant was arranged for free amino acids analysis. The PS-binding amino acid (%) was calculated as follows: CPS=(C0-Csup)/C0, where C0 is amino acid content without treatment; Csup is amino acid content in supernatant treated with PS nanoplastics.

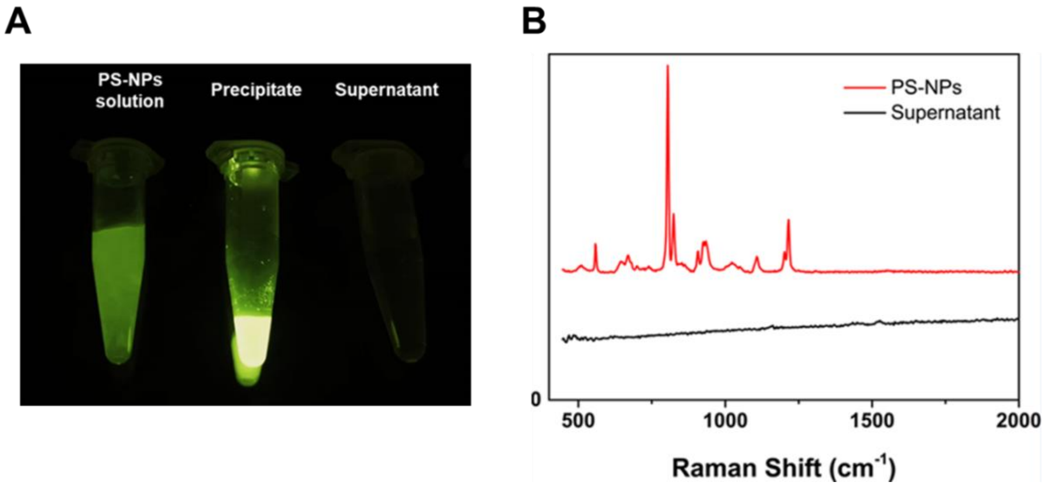

Figure S1. Leaching detection of fluorescent agent or additives in fluorescently-labeled PS nanoparticles. (A) Fluorescence image of PS-NPs solution (stored in lab for one year), precipitate and supernatant. The solution sample (1 mL) was centrifuged at 10000 × g for 5 min, then the precipitate and supernatant were observed at 505 nm excitation. (B) Raman spectra of PS-NPs solution and its supernatant.

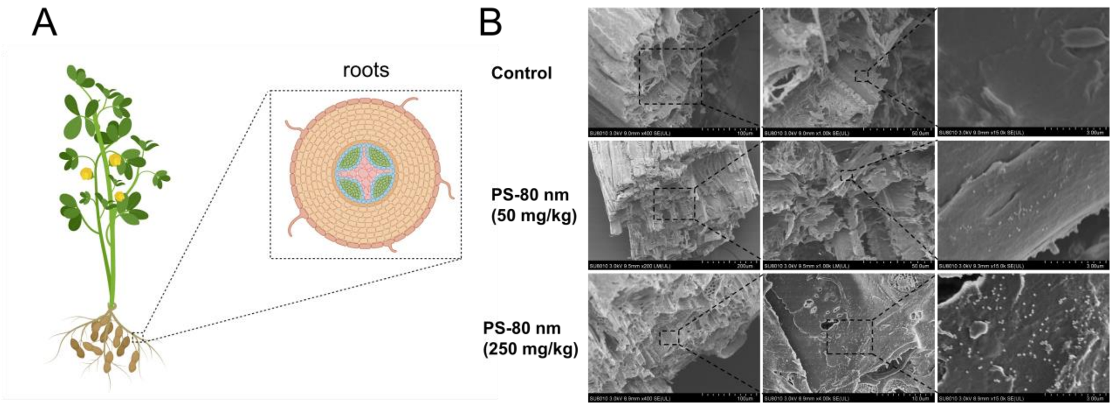

Figure S2. Localization of 80 nm PS bead in the root of peanut. (A) Schematic diagram of the cross section of the peanut root. (B) SEM images of 80 nm PS bead in the root with different treatments.

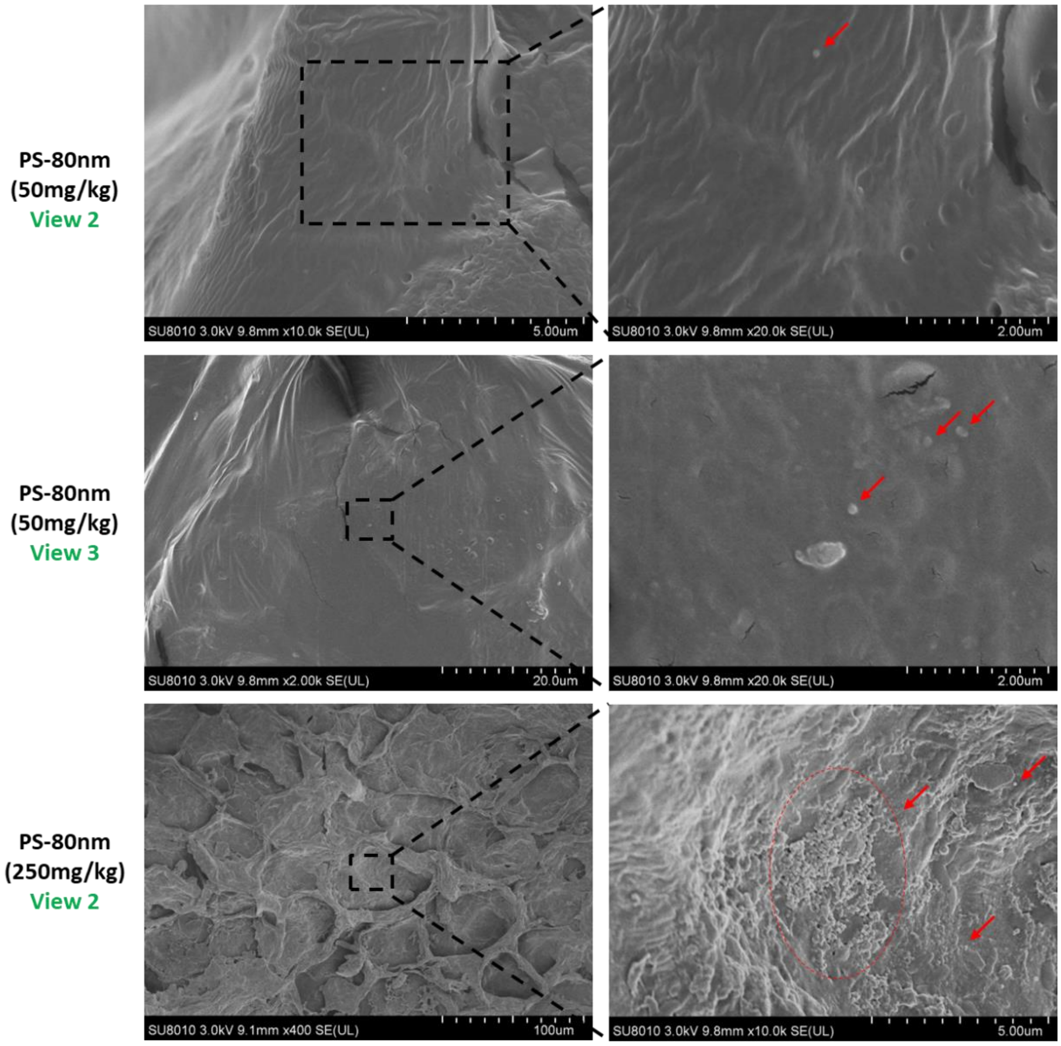

Figure S3. SEM images of 80 nm PS bead localized in the peanut grain under different treatment of PS nanoplastics (in different SEM views). Red arrows indicate nanoplastics.

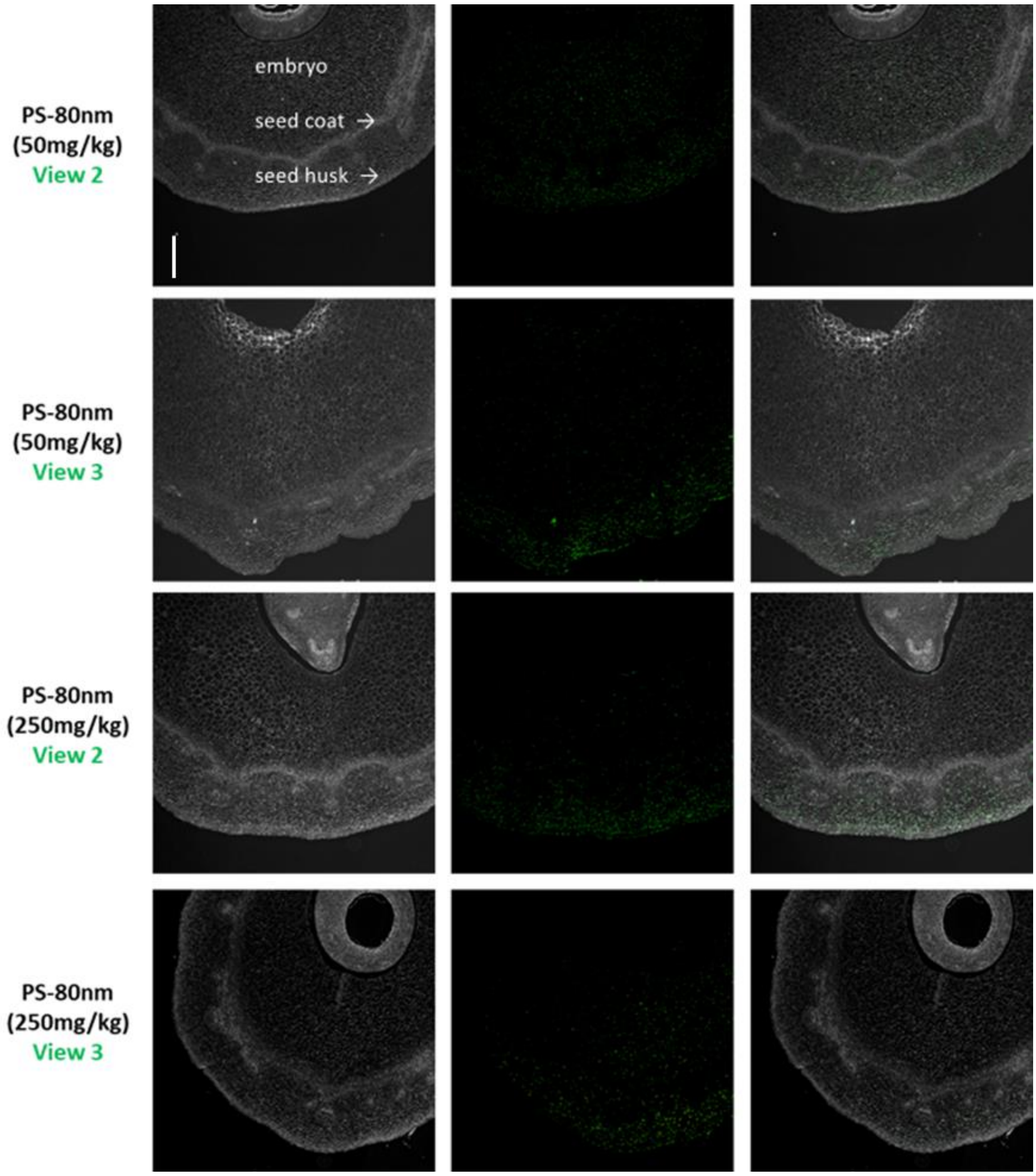

Figure S4. Fluorescence images of accumulation of fluorescently labelled PS beads (80 nm) in peanut grains (in different microscopic field). Scale bars, 500 μm.

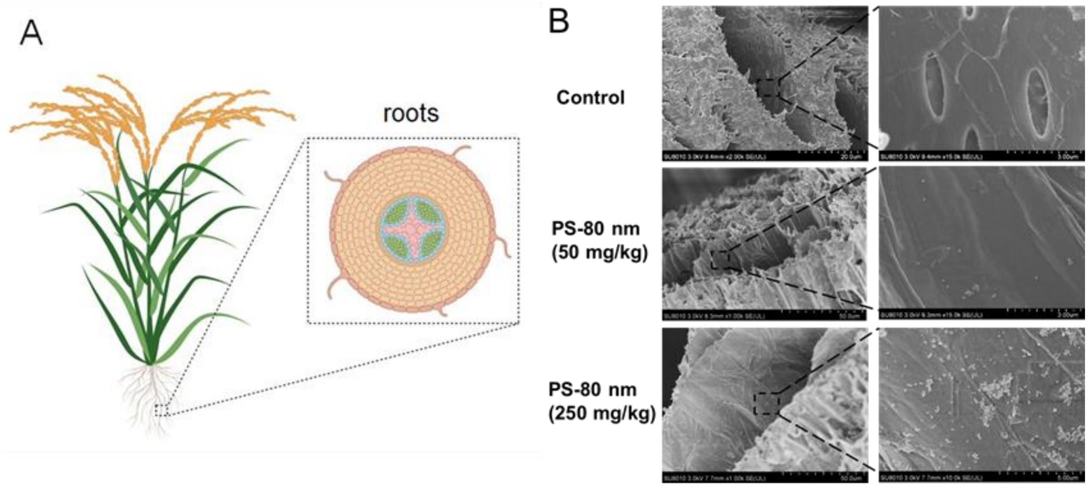

Figure S5. Localization of 80 nm PS bead in the root of rice. (A) Schematic diagram of the cross section of the rice root. (B) SEM images of 80 nm PS bead in the root with different treatments.

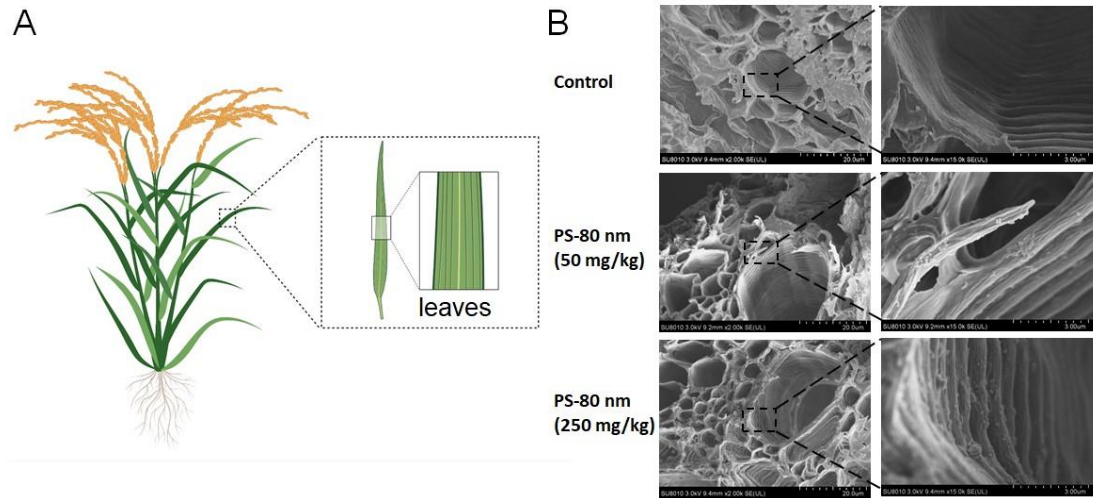

Figure S6. Localization of 80 nm PS bead in the leaves of rice. (A) Schematic diagram of the cross section of the rice leaves. (B) SEM images of 80 nm PS bead in the leaves with different treatments.

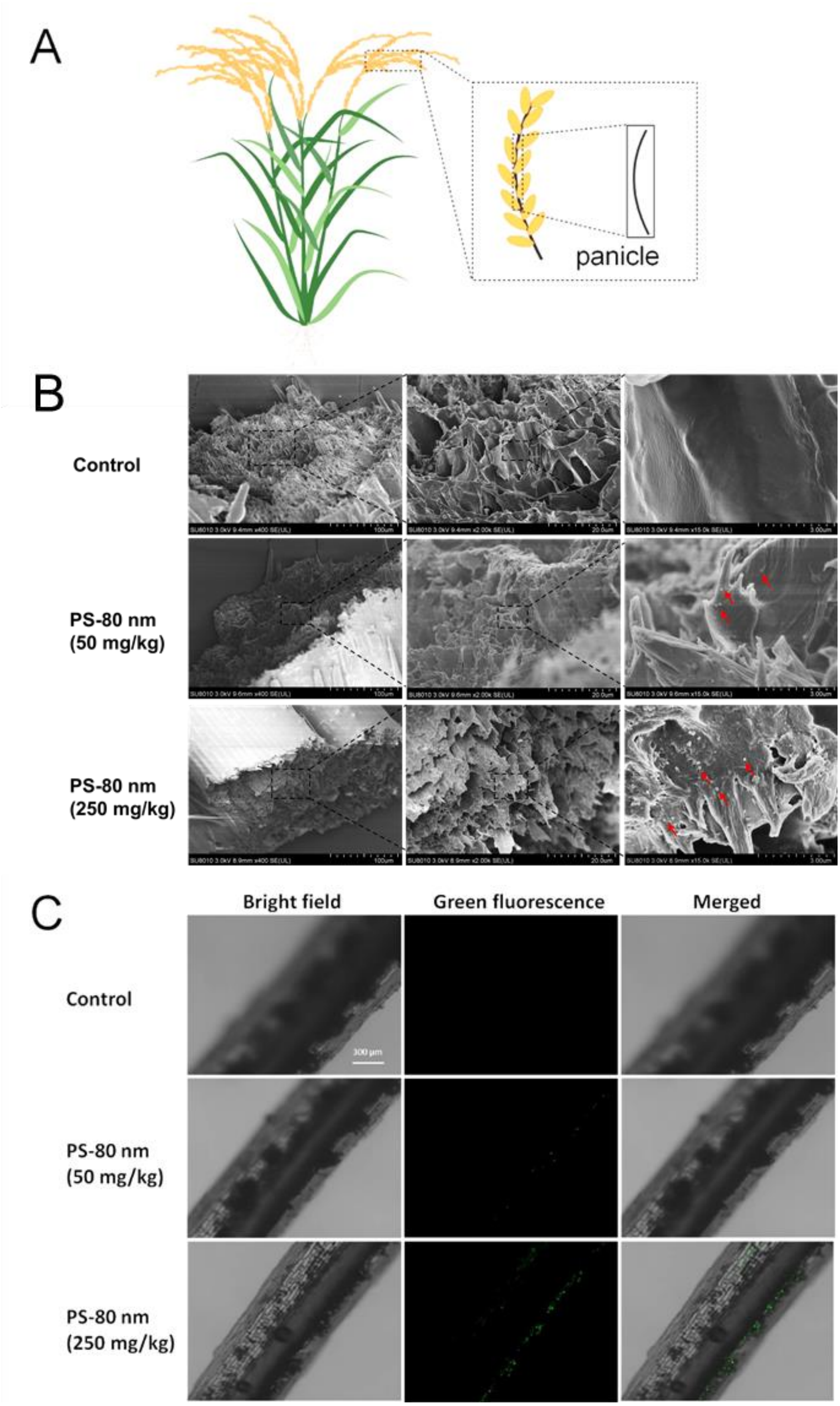

Figure S7. Localization of 80 nm PS bead in the panicle of rice. (A) Schematic

diagram of spike section of the rice plant. (B) SEM images of 80 nm PS bead in the spike at different treatments. Red arrows indicate nanoplastics. (C) Bright field images showing transverse sections of a panicle from a rice plant treated with 80 nm fluorescently labelled PS beads. Green fluorescence images of peanut grain transverse sections were observed using a laser confocal microscope to detect the fluorescently labelled PS beads. Scale bars, 300 μm.

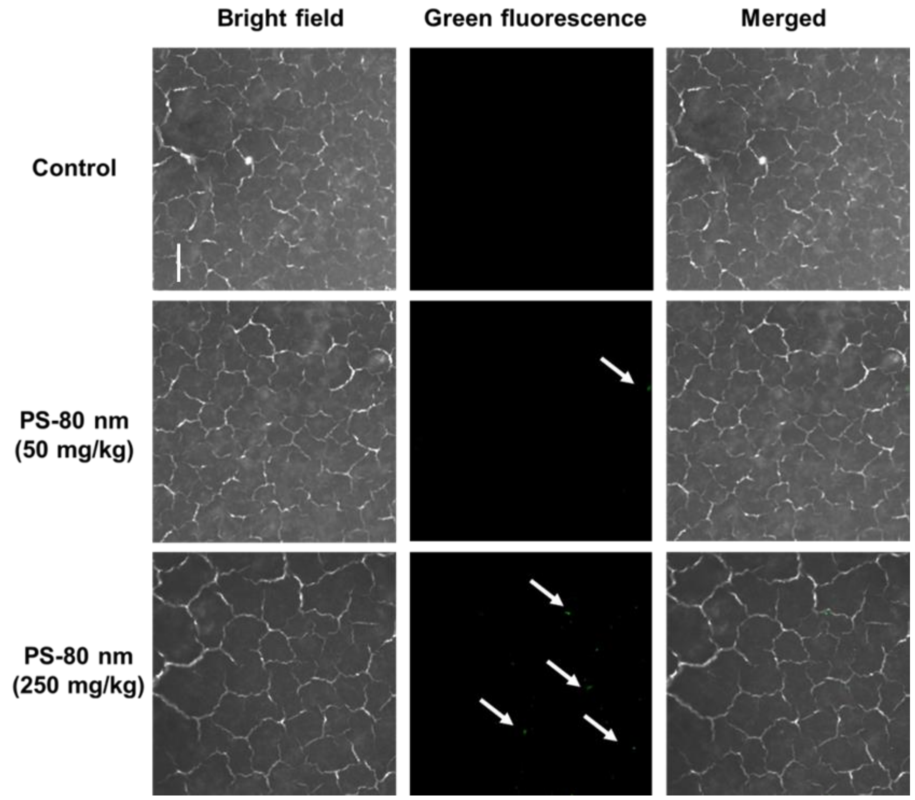

Figure S8. Localization of 80 nm PS bead in the grouting period of a rice grains at different treatments. White arrows indicate nanoplastics. Bright field images showing transverse sections of grain from the grouting period of a rice plant treated with 80 nm fluorescently labelled PS beads. Green fluorescence images of transverse sections of rice grain were observed using a laser confocal microscope to detect the fluorescently labelled PS beads. Scale bars, 300 μm.

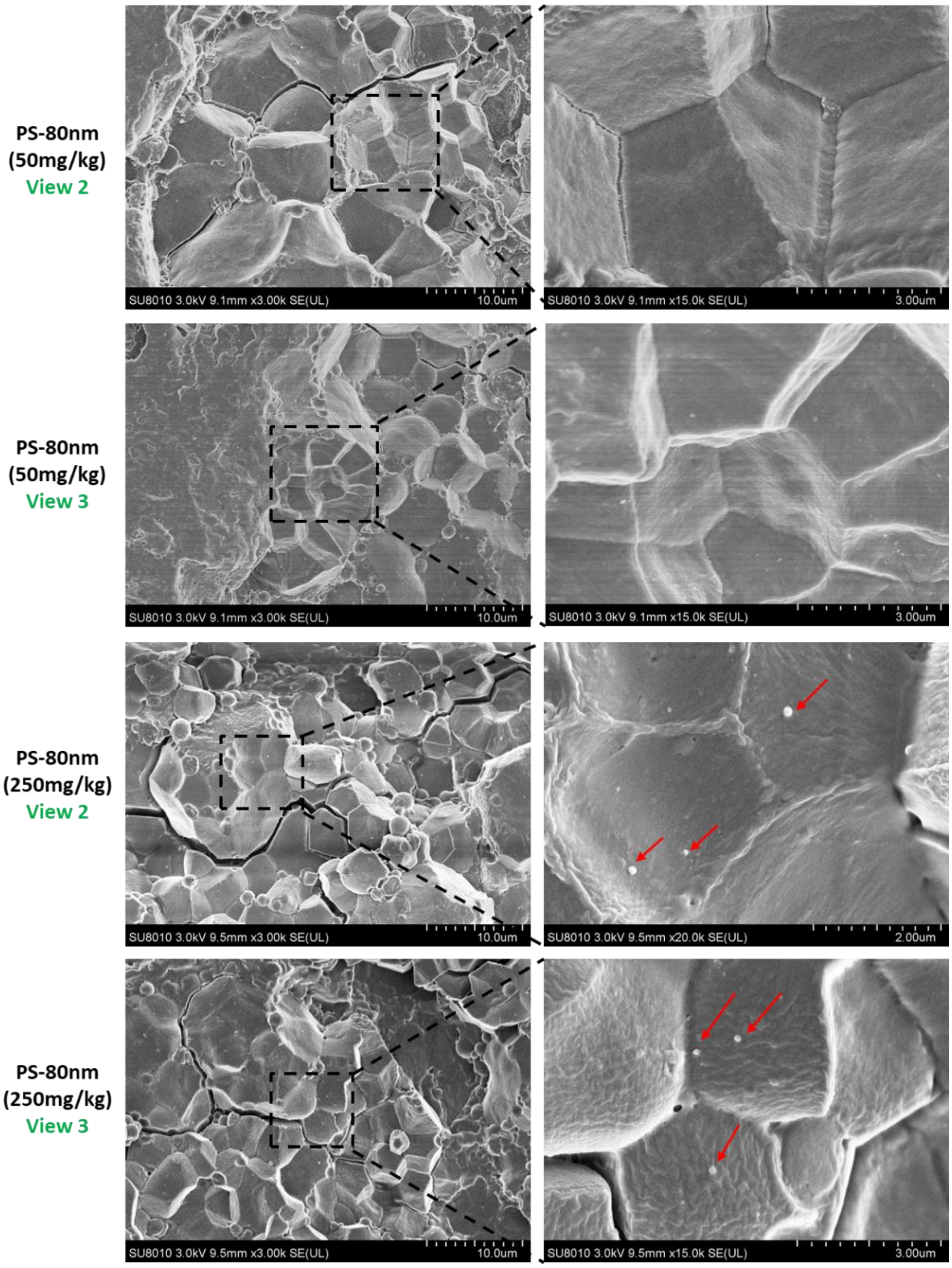

Figure S9. SEM images of 80 nm PS bead localized in rice grains (in different SEM view). Red arrows indicate nanoplastics.

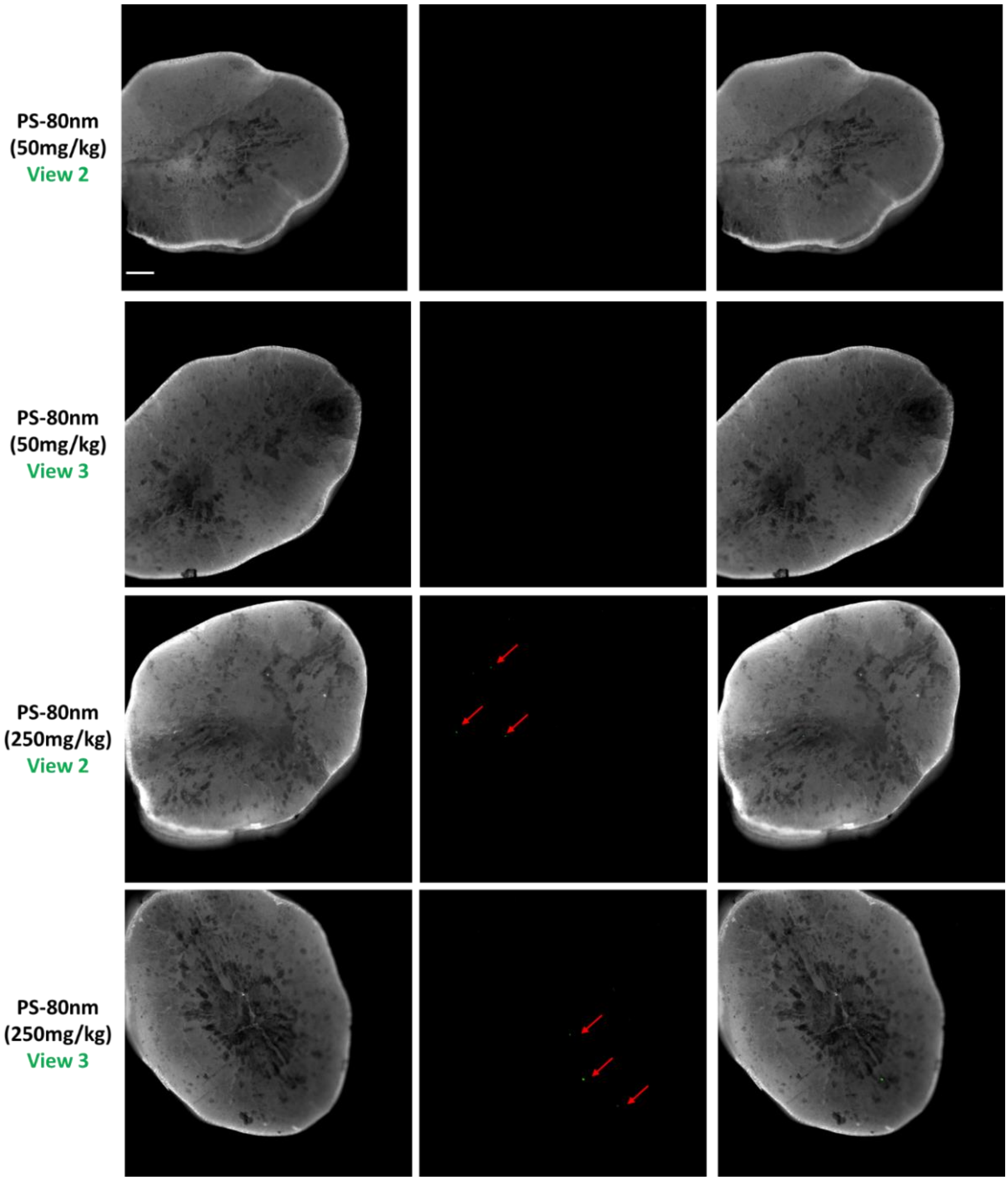

Figure S10. Fluorescence images of the fluorescently labelled PS beads (80 nm) in rice grains (in different microscopic fields). Scale bars, 300 μm. Red arrows indicate fluorescently labelled nanoplastics.

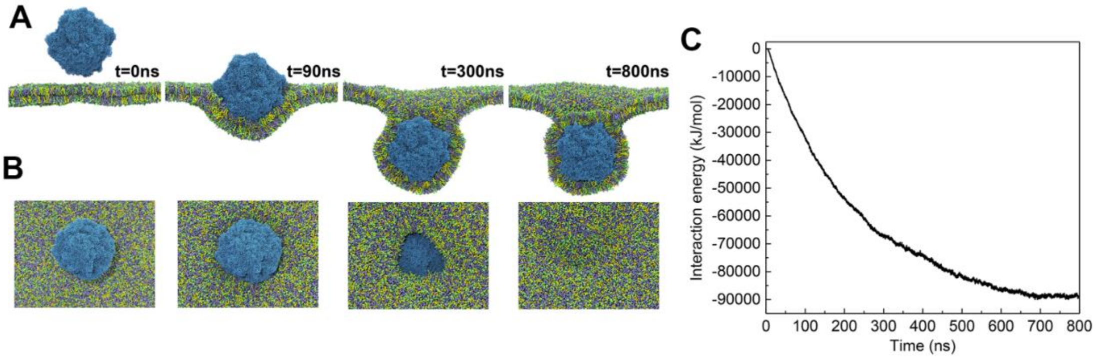

Figure S11. **Molecular dynamics simulations of PS-NPs uptake by the cell membrane bilayers.** (A) Top panel, front view; (B) Bottom panel, top view. Coarse-grained models of POPC, POPE and POPI are colored in purple, yellow and green, respectively. The head group and tail group of lipids are in the sphere and licorice representation, respectively. The PS-NPs are shown in blue spheres. (C) Time evolution of interaction energies (mainly contributed by van der Waals interaction) between PS-NPs and the lipid membranes.

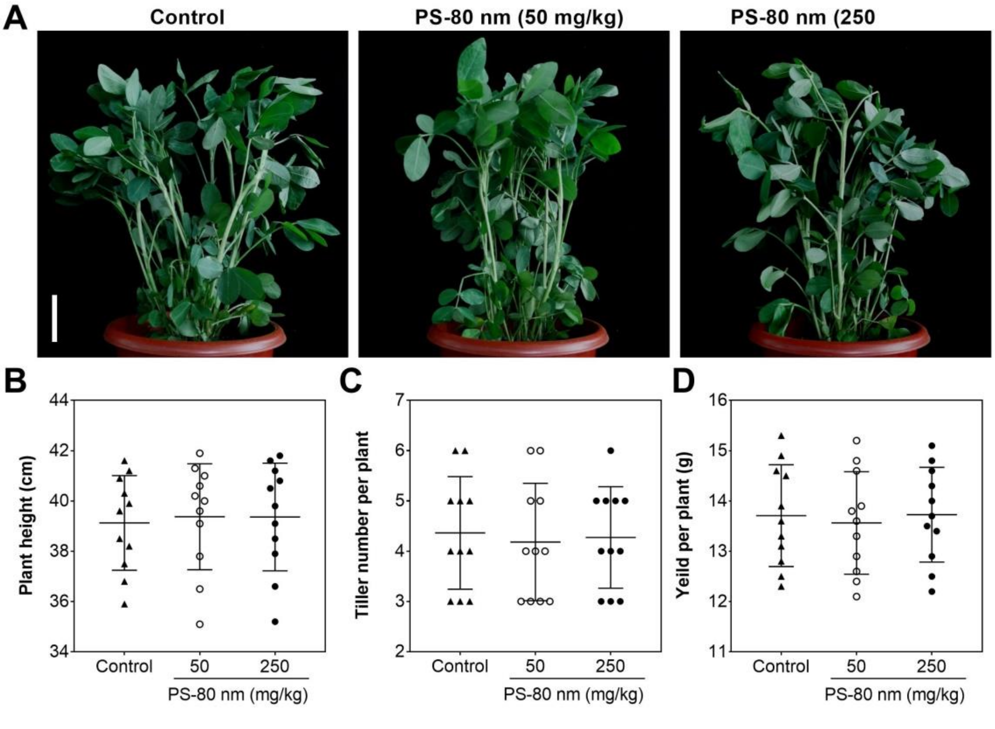

Figure S12. Phenotypic images (A) and physiological parameters (B-D) of peanut plants upon exposure to different treatments of PS nanoparticles. (A) Scale bars, 5 cm. (B-D) plant height, tillering number, and yield per plant, respectively. The analyses were performed independently ten times.

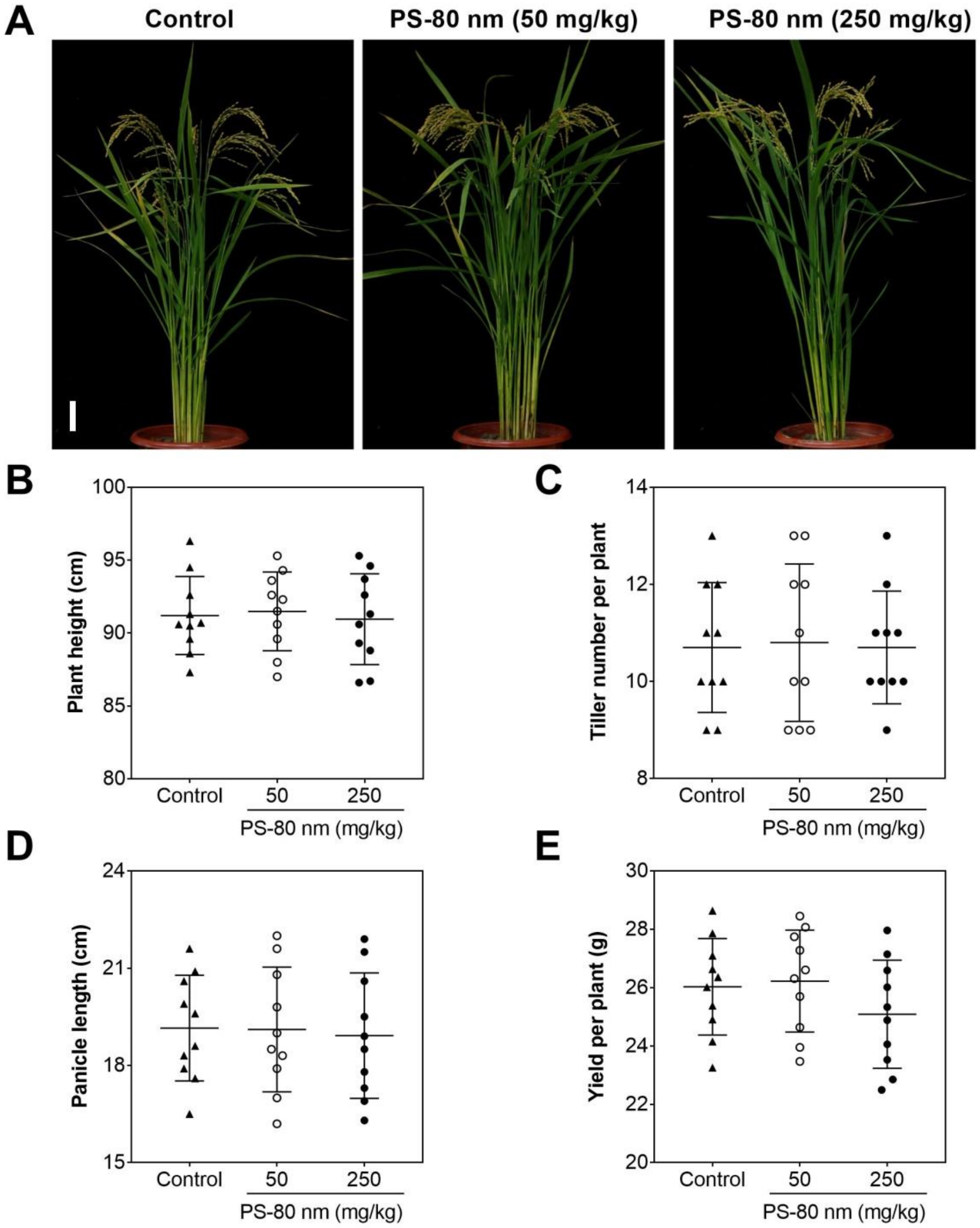

Figure S13. Phenotypic images (A) and physiological parameters (B-D) of rice seedlings upon exposure to different treatments of PS nanoparticles. (A) Scale bars, 5 cm. (B-E) plant height, tillering number per plant, panicle length, and yield per plant, respectively. The analyses were performed independently ten times.

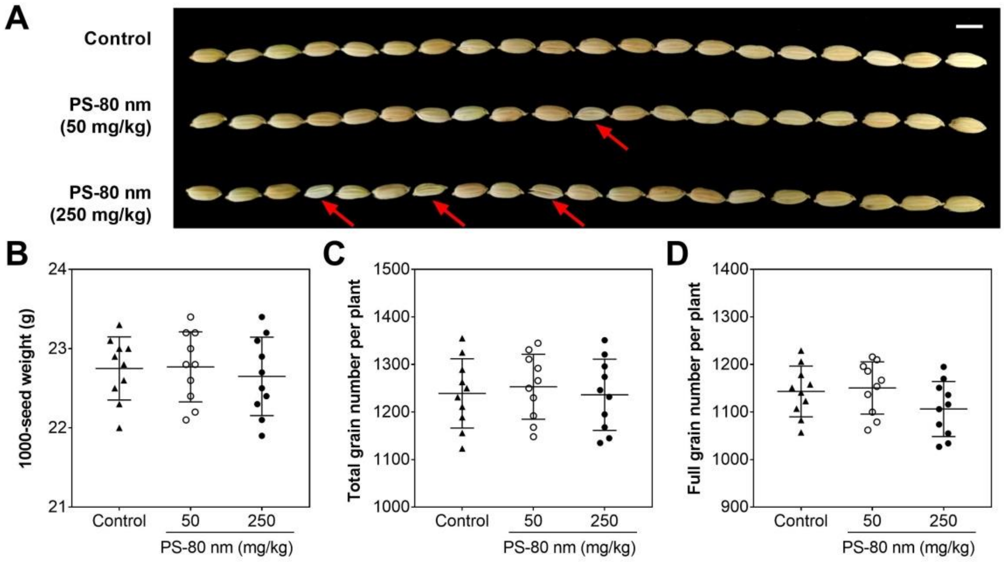

Figure S14. Phenotypic images (A) and physiological parameters (B-D) of rice grains upon exposure to different treatments of PS nanoparticles. (A) Scale bars, 5 mm. (B-D) 1000-grain weight, total grain number per plant, full grain number per plant, respectively. The analyses were performed independently ten times.

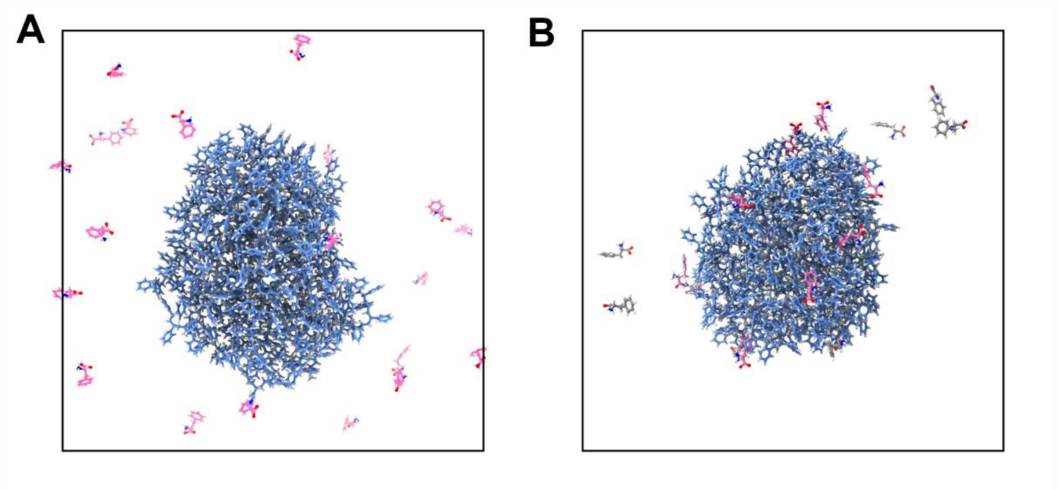

Figure S15. **The initial (A) and final (B) configurations of Phe binding to PS-NPs.** The PS-NPs and Phe are colored by blue and pink, respectively. For clarity, the free amino acids in the final configuration are shown in grey.

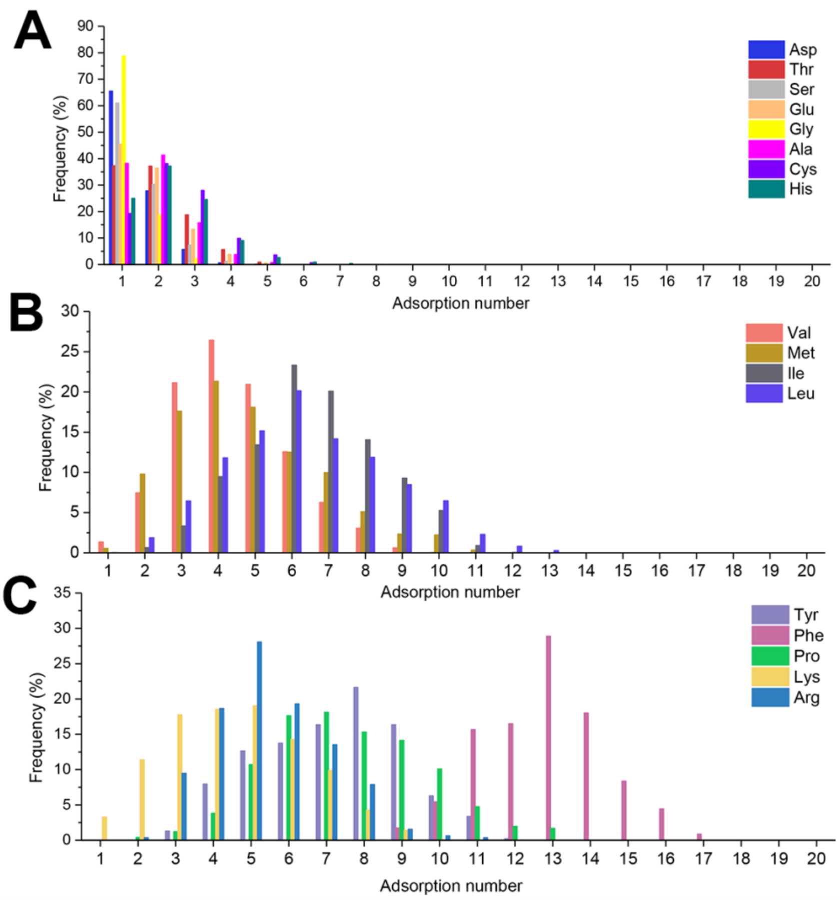

Figure S16. **The distribution of adsorption numbers of amino acids on PS-NPs, and the calculations were based on the last 100 ns trajectory of each system.**

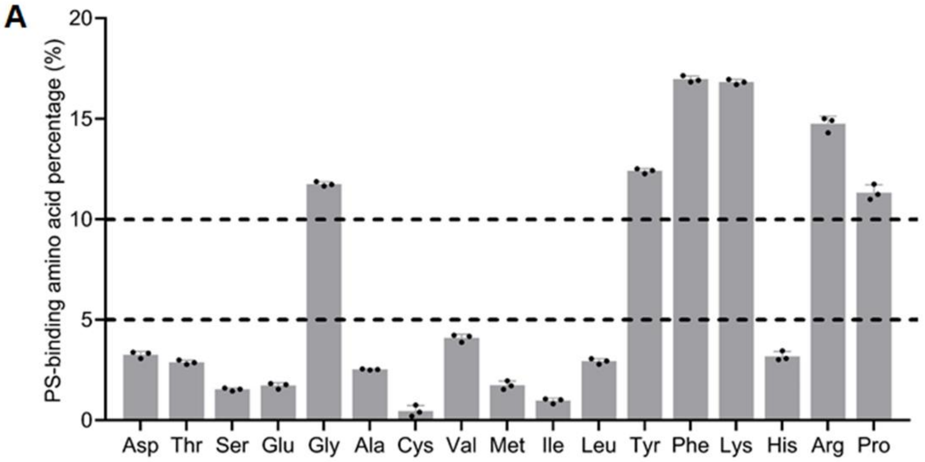

Figure S17. PS-binding amino acid percentage in aqueous solution.

Table S1. Chemical properties of the soil.

Test indicators (g/kg)	Na	Mg	Al	P	K	Ca	Fe	Cu	Zn	pH
**Results**	12.063	12.802	63.122	0.515	23.061	22.66	33.736	0.043	0.077	7.8

Table S2. The relative contents (%) of fatty acids in rice grains.

Fatty Acid (%)	Control	PS-80 nm (50 mg/kg)	PS-80 nm (250 mg/kg)
**C10:0**	0.00147 ± 0.00006 a	0.00139 ± 0.00016 a	0.00132 ± 0.00017 a
**C12:0**	**0.01896 ± 0.00091 a**	**0.01784 ± 0.00042 ab**	**0.01749 ± 0.00010 b**
**C14:0**	0.70848 ± 0.03503 a	0.70147 ± 0.00771 a	0.71724 ± 0.07287 a
**C15:0**	0.04758 ± 0.00120 a	0.04778 ± 0.00072 a	0.04582 ± 0.00237 a
**C16:0**	22.55845 ± 0.45776 a	22.67949 ± 0.41675 a	22.84485 ± 0.16332 a
**C16:1**	0.35931 ± 0.04002 a	0.35725 ± 0.03139 a	0.35764 ± 0.02486 a
**C17:0**	**0.08319 ± 0.00192 a**	**0.08649 ± 0.00324 a**	**0.07697 ± 0.00161 b**
C18:0	5.68050 ± 0.34683 a	5.62980 ± 0.16786 a	5.54969 ± 0.26816 a
**C18:1N9**	39.42905 ± 0.56137 a	39.40454 ± 0.39336 a	39.28282 ± 0.94545 a
**C18:2N6**	27.45377 ± 0.33124 a	27.48066 ± 0.27805 a	27.50612 ± 0.50733 a
**C18:3N3**	0.02023 ± 0.00268 a	0.01868 ± 0.00229 a	0.01924 ± 0.00164 a
**C20:0**	1.52745 ± 0.07981 a	1.49111 ± 0.07900 a	1.52143 ± 0.06968 a
**C20:1N9**	**0.73262 ± 0.00482 a**	**0.71209 ± 0.00684 ab**	**0.69477 ± 0.01747 b**
**C20:2N6**	**0.03933 ± 0.00219 a**	**0.03978 ± 0.00166 a**	**0.03193 ± 0.00254 b**
**C21:0**	0.02036 ± 0.00102 a	0.02020 ± 0.00038 a	0.02205 ± 0.00147 a
**C22:0**	0.43656 ± 0.01513 a	0.43696 ± 0.01608 a	0.43960 ± 0.03576 a
**C22:1N9**	0.07923 ± 0.01109 a	0.07800 ± 0.00425 a	0.07730 ± 0.02341 a
**C24:0**	0.80345 ± 0.03817 a	0.79647 ± 0.02778 a	0.79371 ± 0.02341 a

Different letters indicate the significant differences at $P < 0.05$.

Table S3. The relative contents (%) of fatty acids in peanut grains.

Fatty Acid (%)	Control	PS-80 nm (50 mg/kg)	PS-80 nm (250 mg/kg)
**C10:0**	**0.02094 ± 0.00097 a**	**0.02145 ± 0.00152 a**	**0.02653 ± 0.00363 b**
**C12:0**	0.01037 ± 0.00116 a	0.01218 ± 0.00072 a	0.01012 ± 0.00053 a
**C14:0**	0.04992 ± 0.00019 a	0.04751 ± 0.00085 b	0.05008 ± 0.00046 a
**C15:0**	**0.01009 ± 0.00092 a**	**0.01187 ± 0.00127 a**	**0.02035 ± 0.00115 b**
**C16:0**	**11.27503 ± 0.21922 a**	**11.22606 ± 0.17303 a**	**12.29792 ± 0.05793 b**
**C16:1**	**0.07353 ± 0.00024 a**	**0.07471 ± 0.00505 a**	**0.05048 ± 0.00236 b**
**C17:0**	0.00998 ± 0.00097 a	0.00794 ± 0.00063 b	0.00977 ± 0.00175 a
**C18:0**	3.82685 ± 0.09637 a	4.00081 ± 0.18912 a	3.87700 ± 0.04770 a
**C18:1N9**	46.17546 ± 0.13170 a	46.10724 ± 0.37102 a	46.36021 ± 0.18445 a
**C18:2N6**	32.54571 ± 0.22647 a	32.51876 ± 0.17914 a	31.36266 ± 0.09232 a
**C18:3N3**	**0.07989 ± 0.00158 a**	**0.07240 ± 0.00152 b**	**0.06935 ± 0.00235 b**
**C20:0**	1.60509 ± 0.03375 a	1.59645 ± 0.03289 a	1.60726 ± 0.07363 a
**C20:1N9**	0.84406 ± 0.02902 a	0.84710 ± 0.02833 a	0.86164 ± 0.05591 a
**C20:2N6**	0.02885 ± 0.00381 a	0.02418 ± 0.00148 a	0.02075 ± 0.00144 a
**C21:0**	0.02257 ± 0.00136 b	0.01632 ± 0.00364 c	0.03116 ± 0.00348 a
**C22:0**	2.24992 ± 0.01991 a	2.25813 ± 0.02596 a	2.17707 ± 0.06644 a
**C22:1N9**	0.07248 ± 0.00240 a	0.07616 ± 0.00130 a	0.07150 ± 0.00349 a
**C24:0**	1.09926 ± 0.00509 a	1.08073 ± 0.01021 a	1.09615 ± 0.09671 a

Different letters indicate the significant differences at $P < 0.05$.

Table S4. The total content of amino acids in rice grains.

Amino Acid (g/kg)	Control	PS-80 nm (50 mg/kg)	PS-80 nm (250 mg/kg)
**Asp**	6.55 ± 0.14 a	6.47 ± 0.24 a	6.49 ± 0.20 a
**Thr**	2.86 ± 0.11 a	3.06 ± 0.05 a	3.05 ± 0.11 a
**Ser**	3.44 ± 0.15 a	3.40 ± 0.06 a	3.41 ± 0.21 a
**Glu**	**14.88 ± 0.15 a**	**14.36 ± 0.11 b**	**13.64 ± 0.03 c**
**Gly**	5.63 ± 0.07 a	5.43 ± 0.15 a	5.45 ± 0.2 a
**Ala**	3.72 ± 0.07 a	3.60 ± 0.10 a	3.55 ± 0.10 a
**Cys**	1.23 ± 0.01 a	1.27 ± 0.06 a	1.27 ± 0.06 a
**Val**	4.46 ± 0.06 a	4.36 ± 0.14 a	4.24 ± 0.07 a
**Met**	**1.89 ± 0.01 a**	**1.77 ± 0.08 b**	**1.78 ± 0.09 b**
**Ile**	**2.76 ± 0.03 a**	**2.75 ± 0.07 a**	**2.32 ± 0.09 b**
**Leu**	6.59 ± 0.24 a	6.59 ± 0.22 a	6.49 ± 0.08 a
**Tyr**	**1.54 ± 0.04 a**	**1.51 ± 0.05 a**	**1.34 ± 0.05 b**
**Phe**	**3.92 ± 0.08 a**	**3.83 ± 0.06 a**	**3.35 ± 0.07 b**
**Lys**	**3.28 ± 0.14 a**	**3.35 ± 0.06 a**	**2.93 ± 0.07 b**
**His**	**2.81 ± 0.12 a**	**2.79 ± 0.04 a**	**2.45 ± 0.06 b**
**Arg**	**6.03 ± 0.19 a**	**5.98 ± 0.17 a**	**5.46 ± 0.17 b**
**Pro**	3.81 ± 0.04 a	3.85 ± 0.08 a	3.70 ± 0.05 a

SUM	**75.40 ± 1.59 a**	**73.38 ± 1.66 ab**	**70.92 ± 1.48 b**

Different letters indicate the significant differences at *P* < 0.05.

Table S5. The total content of amino acids in peanut grains.

Amino Acid (g/kg)	Control	PS-80 nm (50 mg/kg)	PS-80 nm (250 mg/kg)
**Asp**	18.51 ± 1.36 a	18.35 ± 0.97 a	18.28 ± 1.41 a
**Thr**	3.02 ± 0.16 a	2.88 ± 0.14 a	3.15 ± 0.25 a
**Ser**	7.69 ± 0.42 a	7.53 ± 0.27 a	7.66 ± 0.40 a
**Glu**	29.02 ± 1.02 a	28.88 ± 1.27 a	28.79 ± 0.43 a
**Gly**	9.11 ± 0.52 a	9.11 ± 0.61 a	9.28 ± 0.18 a
**Ala**	6.57 ± 0.27 a	6.64 ± 0.31 a	6.58 ± 0.44 a
**Cys**	1.90 ± 0.29 a	1.84 ± 0.20 a	1.79 ± 0.19 a
**Val**	7.95 ± 0.47 a	7.86 ± 0.48 a	7.95 ± 0.47a
**Met**	1.21 ± 0.08 a	1.23 ± 0.11 a	1.26 ± 0.15 a
**Ile**	11.78 ± 0.29 a	11.53 ± 0.09 a	11.36 ± 0.44 a
**Leu**	**25.87 ± 0.73 a**	**24.98 ± 0.75 ab**	**23.99 ± 0.75 b**
**Tyr**	**10.09 ± 0.50 a**	**10.14 ± 0.47 a**	**9.12 ± 0.51 b**
**Phe**	**13.15 ± 0.95 a**	**13.13 ± 1.00 a**	**12.13 ± 0.8 b**
**Lys**	**17.43 ± 1.36 a**	**17.13 ± 0.46 a**	**15.45 ± 0.53 b**
**His**	7.45 ± 1.08 a	6.85 ± 0.81 a	7.81 ± 0.36 a
**Arg**	**24.08 ± 1.89 a**	**23.6 ± 1.14 a**	**22.02 ± 0.42 b**
**Pro**	12.11 ± 0.48 b	11.24 ± 0.25 c	13.59 ± 0.11 a
**SUM**	206.96 ± 11.68 a	202.91 ± 8.81 a	200.22 ± 7.61 a

Different letters indicate the significant differences at *P* < 0.05.

Reference

1. Rossi, G.; Monticelli, L.; Puisto, S. R.; Vattulainen, I.; Ala-Nissila, T., Coarse-graining polymers with the martini force-field: Polystyrene as a benchmark case. *Soft Matter* **2011**, *7 (2)*, 698-708.
2. Yoshida, H.; Tanigawa, T.; Kuriyama, I.; Yoshida, N.; Tomiyama, Y., *et al*., Variation in fatty acid distribution of different acyl lipids in rice (oryza sativa l.) brans. *Nutrients* **2011**, *3 (4)*, 505-14.
3. Zhang, C.; Hicks, G. R.; Raikhel, N. V., Molecular composition of plant vacuoles: Important but less understood regulations and roles of tonoplast lipids. *Plants (Basel)* **2015**, *4 (2)*, 320-33.
4. Pogozheva, I. D.; Armstrong, G. A.; Kong, L.; Hartnagel, T. J.; Carpino, C. A., *et al*., Comparative molecular dynamics simulation studies of realistic eukaryotic, prokaryotic, and archaeal membranes. *J. Chem. Inf. Model*. **2022**, *62 (4)*, 1036-1051.
5. Selvam, B.; Yu, Y. C.; Chen, L. Q.; Shukla, D., Molecular basis of the glucose transport mechanism in plants. *ACS Cent. Sci*. **2019**, *5 (6)*, 1085-1096.

6. Wassenaar, T. A.; Ingolfsson, H. I.; Bockmann, R. A.; Tieleman, D. P.; Marrink, S. J., Computational lipidomics with insane: A versatile tool for generating custom membranes for molecular simulations. *J. Chem. Theory Comput*. **2015**, *11 (5)*, 2144-55.
7. Parrinello, M.; Rahman, A., Polymorphic transitions in single crystals a new molecular dynamics method. *J. Appl. Phys*. **1981**, *52 (12)*, 7182-7190.
8. Bussi, G.; Donadio, D.; Parrinello, M., Canonical sampling through velocity rescaling. *J. Chem. Phys*. **2007**, *126 (1)*, 014101.
9. Marrink, S. J.; Risselada, H. J.; Yefimov, S.; Tieleman, D. P.; de Vries, A. H., The martini force field: Coarse grained model for biomolecular simulations. *J. Phys. Chem. B* **2007**, *111 (27)*, 7812-24.
10. De Jong, D. H.; Baoukina, S.; Ingólfsson, H. I.; Marrink, S. J., Martini straight: Boosting performance using a shorter cutoff and gpus. *Comput. Phys. Commun*. **2016**, *199*, 1-7.
11. Hess, B.; Kutzner, C.; van der Spoel, D.; Lindahl, E., Gromacs 4: Algorithms for highly efficient, load-balanced, and scalable molecular simulation. J. Chem. *Theory Comput*. **2008**, 4 (3), 435-447.
12. Humphrey, W.; Dalke, A.; Schulten, K., Vmd: Visual molecular dynamics. J. Mol. *Graphics Modell*. **1996**, 14 (1), 33-38.
